# Multi-under-Actuated Unmanned Surface Vessel Coordinated Path Tracking

**DOI:** 10.3390/s20030864

**Published:** 2020-02-06

**Authors:** Zefang Li, Zhong Liu, Jianqiang Zhang

**Affiliations:** College of Weaponry Engineering, Naval University of Engineering, Wuhan 430033, China; liuz531@163.com (Z.L.); zhangjq_007@163.com (J.Z.)

**Keywords:** under-actuated unmanned surface vehicles (USV), multi-USV control, integral line-of-sight guidance, path tracking

## Abstract

Multi-under-actuated unmanned surface vehicles (USV) path tracking control is studied and decoupled by virtue of decentralized control. First, an improved integral line-of-sight guidance strategy is put forward and combined with feedback control to design the path tracking controller and realize the single USV path tracking in the horizontal plane. Second, graph theory is utilized to design the decentralized velocity coordination controller for USV formation, so that multiple USVs could consistently realize the specified formation to the position and velocity of the expected path. Third, cascade system theory and Lyapunov stability are used to respectively prove the uniform semi-global exponential stability of single USV path tracking control system and the global asymptotic stability and uniform local exponential stability of coordinated formation system. At last, simulation and field experiment are conducted to analyze and verify the advancement and effectiveness of the proposed algorithms in this paper.

## 1. Introduction

Unmanned surface vehicle (USV) is a small intelligent mission platform on the water surface, and has such advantages as small size, low cost, high speed, intelligence, small radar cross section, and no personal injury or death [1]. In the military domain, it could realize flexible deployment and combat. Equipped with different mission modules, it could complete a variety of missions such as information gathering, surveillance and reconnaissance, target strike, minesweeping, anti-submarine, and damage assessment. USV could set out from the port and fulfill its mission as scheduled, or sail with combat warships to complete the mission independently when combat warships are not suitable for the mission or in a dangerous sea area. Therefore, it has got great attention from navies around the world since it could greatly expand the range of naval combat, and become an “amplifier” for own combat effectiveness [2]. In the civil domain, USV could be used for hydro-geological survey, search and rescue, relay communication, etc., and it is a key node for integrating low-altitude unmanned aerial vehicle and underwater robot across network. Therefore, its application has a bright prospect [3].

The study of USV coordinated formation control started quite late, but has great potential for actual application. In the civil domain, it could be performed to not only coordinate the hydrologic and topographic surveys in a large sea area, but also complete the pavement and patrol of subsea pipeline. In the military domain, it could be conducted for large minefield detection and clearing, and tactical formation could be employed to block the enemy warship or port, and enhance the combat efficiency and the success rate of enemy target strike. Therefore, it is of great significance.

### 1.1. USV Control

With regard to USV path tracking control, Børhaug et al. (2011) put forward the integral line-of-sight (ILOS) guidance control law as a compensation for the environmental disturbance, and demonstrated the stability of system [4]. John et al. (2013) introduced an input-output feedback linearization control approach based on neural network for anti-lock braking system (ABS) control, which overcame the failure of traditional PID (Proportion Integral Differential), slide mode, and feedback linearization control to cope with the strong nonlinearity of friction between tyre and ground and the uncertainty of vehicle kinetic parameters. Additionally, the differences were verified in a laboratory test. However, the algorithm was coupled with the model, and could not expand into other control fields [5]. In 2014, Caharija et al. designed the global K-exponential straight path tracking controller and ocean current observer based on coordinate transformation, but did not take into account the common problem of curved path tracking control [6]. Lekkas et al. (2014) studied path tracking control while considering the disturbance caused by external factors such as ocean current to the USV kinematic model, but ignored the asymmetry of USV model [7]. Moreover, Fredriksen et al. (2014) designed the path tracking ILOS guidance algorithm based on relative velocity model, and proved the global K-exponential stability of control system, but only straight path tracking was realized [8]. Blažic (2014) put forth the periodic controller based on the Lyapunov theory for two differential drive wheeled robots, and verified its global convergence. However, the study focused on wheeled robots, so that the model was much different from that for USV. Additionally, the study did not take into account the influence of external disturbance [9]. In 2015, Tian et al. designed the straight tracking controller based on the line-of-sight guidance strategy and anti-saturation PID control algorithm, but overlooked the influence of external disturbance [10]. Takács et al. (2015) analyzed and generalized the main components, practical difficulties and challenges of remote surgical system, and proposed some corresponding solutions. Based on their discussion, a remote surgical system control approach was proposed for integrated modelling, but it was designed for surgical robot, so that the control model was limited to certain applications [11]. In 2018, Pozna et al. introduced an economical and effective approach for designing the nonlinear state and space control system, which was general and applicable to LTI (Linear Time-Invariant), LTV (Linear Time-Varying), LPV (Linear Parameter-Varying), and q-LPV (quasi-Linear Parameter-Varying) systems. Through the cart-pendulum system in a laboratory, they verified the effectiveness of the approach. However, the approach depends much on the model, so that it is not applicable to the control applications using the model with uncertainties. In the meanwhile, the approach is quite theoretical, and not meaningful to engineering applications [12].

At present, most USV path tracking algorithms including those used in the above references are designed with simplified system and numerical analysis. In their design, it is assumed that USV is longitudinally and laterally symmetrical. All the models are strictly diagonal, and employ inertial coefficient matrix and damping coefficient matrix. However, USV often has a structure of lateral symmetry but longitudinal asymmetry. Therefore, modeling results in very large errors. Algorithms are designed with known disturbance, so that the disturbance caused by ocean current to the USV kinematic model is overlooked in most references, but regarded as the disturbance to the USV kinetic model together with wind, wave and un-modelled dynamics. As a matter of fact, ocean current exerts only kinematic effects on the USV maneuvering motion, and causes USV drifting to change its velocity and position and make it deviate from the planned route and heading. In the meanwhile, some references rely on much prior knowledge of single structure, so that the models are less adaptive to environment. Error direct feedback disturbance observer is used in the design to observe disturbance in a real-time manner, which sacrifices the controllability of system.

With regard to USV formation control, Shojaei et al. (2015) studied the under-actuated USV formation control under external disturbance and model parameter perturbation. After firstly designing a formation coordination controller based on the leader-follower mode, they utilized state feedback in the design of single USV tracking controller and introduced generalized saturation function to effectively prevent the possible oversaturation caused by actuating mechanism. After that, neural network was utilized to online approach the uncertain terms caused by external disturbance, and eventually Lyapunov stability theory was employed to prove the uniform semi-global stability of closed-loop control system. However, leader’s breakdown may lead to the collapse of the formation so that the leader-follower mode is not applicable to practical engineering applications [13]. In 2016, Shojaei further utilized multi-layer neural network and adaptation technology to perform the real-time estimation and compensation for the model perturbation caused by external disturbance on the basis of Reference [13], so as to guarantee the stability of formation controller. In the end, Lyapunov theory was taken as the basis to prove the uniformly ultimate boundedness for all the states and signals of control system [14]. In 2017, Nair et al. proposed an autonomous robot formation control algorithm based on artificial potential field path planning and quick adaptive gain nonsingular terminal sliding mode control, and introduced the idea of tolerance control to ensure the control stability of the entire formation during the failure of single entity. The algorithm was proved feasible in the simulation experiment. Nevertheless, they considered only the position of members in the formation, and paid no attention to the tracking accuracy of each USV with regard to the expected path [15]. Xiao et al. (2017) proposed a combination of unmanned aerial vehicle (UAV) and USV for maritime search and rescue. In the combination, UAV could provide a wider range of vision to enhance the situation perceptibility of USV. However, their study focused on the applications using both UAV and USV for search and rescue, and did not further explore the USV control method. Therefore, the combination is not applicable to the problem of USV formation control, but it provides a new approach to the future studies on USV formation control [16]. In 2018, Ghommam et al. designed a robust adaptive USV formation controller based on Lyapunov’s direct and backstepping methods and following the line-of-sight guidance strategy, to cope with the unknown velocity of leader and the constraint of turning angle on the members in the USV formation control. Based on Lyapunov stability theory and finite time theory, they proved that formation tracking error could converge into a small adjacent domain of approximately 0 within the finite time, while comparative simulation experiment was conducted to verify the effectiveness of the algorithm. However, they did not take into account the problem of collision prevention among formation members and the influence of leader’s failure on formation control in the leader-follower mode [17].

Generally, formation control is often achieved using leader-follower method [18], behavior-based method [19], and virtual structure method [20]. In the leader-follower formation control, robots in the formation are appointed to two complementary roles, i.e., leader and follower. A follower tracks the trajectory of the leader. The formation control is realized in this way. This method could simply control the entire formation by specifying the leader’s behavior or trajectory, but the leader does not pay attention to other robots. Therefore, there is not any feedback on the formation in the system. In other words, the leader and followers are independent from each other, and it is not easy for the leader to obtain the feedback on tracking error from followers [21,22,23,24,25]. In the behavior-based formation control, each kind of behavior has a specific objective and mission. When there are several different objectives, it is easy to determine the collective behavior control strategy. Each robot responds based on the position of other robots, so that there is specific formation feedback. Additionally, this method allows distributed control in a real-time manner. However, it could not clearly indicate the principles for local control of collective behavior, and not guarantee the stability of formation control [26,27,28]. Virtual structure method utilizes the virtual structure of rigid object and the fixed positions in the structure to realize formation control. This method could easily determine the formation control strategy and give formation feedback. It is quite intelligent, and guarantees the stability of formation to some extent. However, it requires the motion of the formation in a virtual structure, so that it is less flexible and adaptive [29,30,31]. Moreover, there are also other formation control methods. For instance, artificial potential field [32] could effectively shun obstacles and avoid collisions when there are obstacle constraints. It features easy calculation and convenient real-time control, but it is difficult to design a reasonable potential field function, and involves local extreme value points. Graph theory [33] could be used to represent any formation, and there are some well-developed formal theories such as algebraic graph theory. Nevertheless, it is very complicated to implement the method, so that it is only limited to simulation research. Model predictive control [23] is a method with strong theoretical basis and massive computation, so that it is very difficult to implement, and not applicable to practical engineering.

In the abovementioned formation control methods, coordination control law is designed only to realize the relative position or distance of USVs as expected and maintain a specific formation for mission execution, but no restriction is imposed on the motion track of each USV. In most references, the design of formation controller does not include external disturbance and uncertainties in USV mathematical model. Most references describe the verification of algorithm only by virtue of simulation, but rarely put it into the actual cruising test of USV coordinated formation.

### 1.2. Main Contributions

This paper focuses on formation path tracking in the problem of formation control, and addresses the problem of coordinated path tracking for multiple under-actuated USVs subject to external disturbance. With the decentralized control strategy, the problem of multi-USV coordinated path tracking is decoupled into the design of single USV path tracking controller and multi-USV coordination controller. In this way, each USV is controlled to overcome the influence of external disturbance and realize accurate path tracking while maintaining the expected formation. Hence, this approach is suitable for some engineering applications with higher requirements for the USV formation path tracking accuracy.

Based on Reference [34], this paper first builds an asymmetric kinematic and kinetic model with three degrees of freedom for under-actuated USV subject to external disturbance in the horizontal plane. At present, the nonlinear mathematical model of USV with three degrees of freedom is employed nearly in all references available at home and abroad, so that this model could accurately present the motion of USV in the horizontal plane. In many other works, this model has been also used, but it is simplified for controller design to different degrees. In this paper, this model is employed but not simplified.

Second, integral line-of-sight guidance is mainly employed as a strategy to address the problem of single USV control. The strategy is further improved by changing the fixed headway range to the time-variant adaptive headway range. Time-variant headway range allows more flexible motion of USV. When USV is very far away from the expected path, tracking error is large, but headway range is small, making USV approach the expected path fast. When USV is close to the expected path, tracking error is small, but headway range is large, which could effectively reduce the position error overshoot. Meanwhile, the influence of USV under-actuation, uncertainty and external disturbance is taken into account comprehensively to design the heading and velocity controller of single USV based on the idea of feedback control. In this way, the expected heading given by the guidance system is tracked so that USV could maintain the accurate and stable tracking of the expected path, which resolves the problem of lower path tracking accuracy caused by time-variant disturbance in the traditional ILOS algorithm. As for multi-USV coordination control system, this paper utilizes the graph theory to design the decentralized velocity coordination controller for USV formation. Therefore, each single USV could accurately track the expected path, while the velocity of each USV is controlled in a decentralized manner to quickly realize the USV formation and maintain it in a coordinated and consistent way.

Subsequently, Lyapunov theory is employed to verify the uniform semi-global exponential stability of single path tracking control system, while guidance system and heading and velocity control system are constructed into a cascade system. The theory of cascade system is utilized to prove the global asymptotic stability and uniform local exponential stability of multi-USV coordinated formation control system. Compared with the global K-exponential stability described in most references, this approach offers faster convergence and stronger robustness.

At last, comparative simulation experiment and USV maritime test are conducted to analyze and verify the advancement and effectiveness of the proposed algorithm in this paper.

This paper is organized as follows: Section 1 analyzes and summarizes the current status and shortcomings of the research on single USV and formation control, and explains the research purpose of this article; Section 2 establishes an asymmetric mathematical model with three degrees of freedom subject to external disturbance for under-actuated USV. In order to avoid controller design difficulties, the model is coordinate transformed, and the USV longitudinal error formula is derived by synthesizing the model and the expected path description; Section 3 decouples the USV formation path tracking problem into a single USV path tracking problem and a multi-USV collaborative formation problem. Through analysis and summary, the control goals of the single USV path tracking and multiple USV collaborative formation are presented, which provides a basis for the controller design below; In order to achieve the control goals proposed in Section 3, Section 4 combined the mathematical model and error formula given in Section 2 and designed single USV path tracking controller and multiple USVs collaborative formation controller based on the improved ILOS guidance strategy, feedback linearization method, and graph theory knowledge; Section 5 proved mathematically the stability of the controller designed by Section 4 through cascade theory and Lyapunov theory, that is, the control target proposed by Section 3 was reachable; Section 6 applies the controller designed in Section 3 to simulation experiments and real ship experiments, and compares it with the single USV control algorithm under traditional ILOS guidance strategy and the formation control algorithm without considering model perturbation and external interference. The result chart proves the effectiveness and advancedness of the algorithm in this paper. The effectiveness of the engineering application of the algorithm in this paper is verified by the test of three USV real ships organized in actual sea area. Section 7 summarizes the work of this paper.

## 2. Under-Actuated USV Model and Problem Description

### 2.1. Under-Actuated USV Model

**Figure 1:**
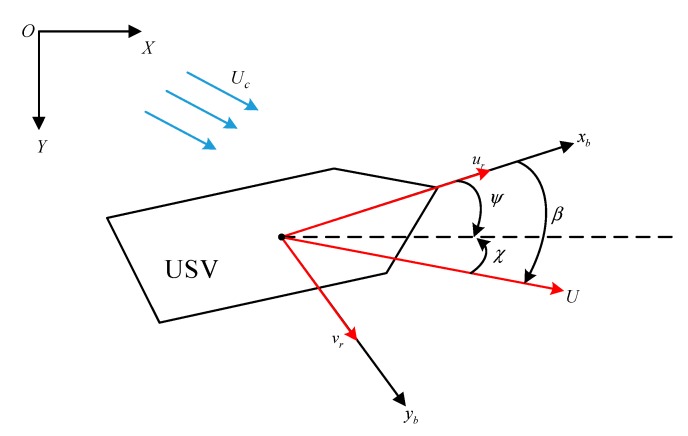
Schematic diagram of unmanned surface vehicles (USV) motion with 3 degrees of freedom (DOF) on the horizontal plane.

Considering the irrotational flow of seawater in the geodetic coordinate system (hereinafter referred to as {i} system), and the constant velocity $V_{c}={\left[V_{x},V_{y},0\right]}^{T}$, there is a resultant velocity $U_{c}=\sqrt{V_{x}^{2}+V_{y}^{2}}$. When the constant is $V_{\max}>0$, we let $V_{\max}\geq U_{c}$ and ${\dot{U}}_{c}=0$, where $V_{c}=\|V_{c}\|$. Hence, the kinematic and kinetic model of under-actuated USV with three degrees of freedom is expressed in the following way:

(1) $$\left\{ \begin{array}{l} \dot{\eta }=J(\psi )v_{r}+V_{c}\\ M{\dot{\upsilon }}_{r}+C(\upsilon_{r})\upsilon_{r}+D(\upsilon_{r})=B\tau \end{array}\right.$$

where $\eta ={\left[x,y,\psi \right]}^{T}$ is the horizontal position and heading angle of USV under the {i} system; $v_{r}={\left[u_{r},v_{r},r\right]}^{T}$ is the longitudinal and transverse linear velocities and the relative velocity of heading angle when USV is under the hull coordinate system (hereinafter referred to as {b} system). The schematic diagram is shown in Figure 1. In the figure, $U=\sqrt{u_{r}^{2}+v_{r}^{2}}$ is the resultant velocity of USV relative motion under the geodetic coordinate system; heading angle ψ is the angle formed by the bow and the OX axis of inertial coordinate system; track angle χ=ψ+β is the angle formed by the velocity U and the OX axis of inertial coordinate system; drift angle β is the angle formed by the surge velocity u and the velocity U of USV, or the angle formed by the track angle and the heading angle; $U_{c}$ is the resultant velocity of irrotational ocean current under the geodetic coordinate system. J(ψ) is the rotation matrix from the {b} system to the {i} system; M is the inertial parameter matrix of under-actuated USV; $C(v)=C_{RB}(v)+C_{A}(v)$ with $C_{RB}(\upsilon_{r})$ and $C_{A}(\upsilon_{r})$ as the respective matrixes of Coriolis force and centripetal force applied by rigid body and hydrodynamics, and $C_{RB}(v)$ is unrelated to the velocities u and v; D(υ) is damping parameter matrix; $\tau ={\left[T_{u},T_{r}\right]}^{T}$ is control input matrix with $T_{u}$ as forward thrust and $T_{r}$ as yawing torque; and vector matrix B is control input configuration matrix. They are defined as follows [34]:

(2) $$J(\psi )=\left[\begin{array}{ccc} \cos\psi & -\sin\psi & 0\\ \sin\psi & \cos\psi & 0\\ 0 & 0 & 1 \end{array}\right]$$

(3) $$M=\left[\begin{array}{ccc} m_{11} & 0 & 0\\ 0 & m_{22} & m_{23}\\ 0 & m_{32} & m_{33} \end{array}\right]$$

(4) $$B=\left[\begin{array}{cc} b_{11} & \;0\\ \;0\; & b_{22}\\ \;0 & b_{32} \end{array}\right]$$

(5) $$C(v)=\left[\begin{array}{ccc} 0 & 0 & -m_{22}v-m_{23}r\\ 0 & 0 & m_{11}u\\ m_{22}v+m_{23}r & -m_{11}u & 0 \end{array}\right]$$

(6) $$D(\upsilon )=\left[\begin{array}{ccc} d_{11} & 0 & 0\\ 0 & d_{22} & d_{23}\\ 0 & d_{32} & d_{33} \end{array}\right]$$

At present, the nonlinear mathematical model of USV with 3 degrees of freedom (DOF) is employed nearly in all references available at home and abroad, so that this model could accurately present the motion of USV in the horizontal plane. In many other works, this model has been also used, but it is simplified for controller design to different degrees. In this paper, this model is employed but not simplified.

### 2.2. Coordinate Transformation

The influence of stock torque on the transverse motion of USV makes it difficult to design the heading and velocity controller. To prevent this influence, the center of USV in the {b} system is transformed into the position of pivoting point.

(7) $$\begin{array}{l} \bar{x}=x+\varepsilon \cos\psi \\ \bar{y}=y+\varepsilon \sin\psi \\ \bar{\psi }=\psi \\ {\bar{u}}_{r}=u_{r}\\ {\bar{v}}_{r}=v_{r}+\varepsilon r\\ \bar{r}=r \end{array}$$

where $\varepsilon =-\frac{\left(m_{33}b_{22}-m_{23}b_{32}\right)}{\left(m_{22}b_{32}-m_{23}b_{22}\right)}$, and the model is transformed into:

(8) $$\dot{\bar{x}}={\bar{u}}_{r}\cos\bar{\psi }-{\bar{v}}_{r}\sin\bar{\psi }+V_{x}$$

(9) $$\dot{\bar{y}}={\bar{u}}_{r}\sin\bar{\psi }+{\bar{v}}_{r}\cos\bar{\psi }+V_{y}$$

(10) $$\dot{\bar{\psi }}=\bar{r}$$

(11) $${\dot{\bar{u}}}_{r}=F_{u_{r}}({\bar{u}}_{r},{\bar{v}}_{r},\bar{r})+\frac{b_{11}}{m_{11}}T_{u}$$

(12) $${\dot{\bar{v}}}_{r}=X({\bar{u}}_{r})\bar{r}+Y({\bar{u}}_{r})\bar{v}$$

(13) $$\begin{array}{l} \;\dot{\bar{r}}=F_{r}({\bar{u}}_{r},{\bar{v}}_{r},\bar{r})+\;\frac{\left(b_{32}m_{22}-b_{32}m_{23}\right)}{\left(m_{22}m_{33}-m_{23}{}^{2}\right)}T_{r}\\ \; \end{array}$$

where

(14) $$F_{u_{r}}({\bar{u}}_{r},{\bar{v}}_{r},\bar{r})=\frac{m_{22}({\bar{v}}_{r}-\varepsilon \bar{r})\bar{r}+m_{23}{\bar{r}}^{2}}{m_{11}}-\frac{d_{11}{\bar{u}}_{r}}{m_{11}}$$

(15) $$\begin{array}{l} X({\bar{u}}_{r})=\frac{\left(m_{23}^{2}-m_{11}m_{33}){\bar{u}}_{r}+(m_{23}d_{33}-m_{33}d_{23}\right)}{m_{22}m_{33}-m_{23}^{2}}\;+\;\\ \;\frac{\left(m_{11}m_{23}-m_{22}m_{23}){\bar{u}}_{r}+(m_{23}d_{23}-m_{22}d_{33}\right)}{m_{22}m_{33}-m_{23}^{2}}\varepsilon \end{array}$$

(16) $$Y({\bar{u}}_{r})=\frac{\left(m_{22}m_{23}-m_{11}m_{23}){\bar{u}}_{r}+(m_{23}d_{32}-m_{33}d_{22}\right)}{m_{22}m_{33}-m_{23}^{2}}$$

(17) $$\begin{array}{l} F_{r}({\bar{u}}_{r},{\bar{v}}_{r},\bar{r})=\\ \frac{(m_{11}m_{23}-m_{22}m_{23}){\bar{u}}_{r}\bar{r}+(m_{23}d_{23}-m_{22}d_{33})\bar{r}}{m_{22}m_{33}-m_{23}^{2}}\;+\\ \frac{\left(m_{11}m_{22}-m_{22}^{2}){\bar{u}}_{r}+(m_{23}d_{22}-m_{22}d_{32}\right)}{m_{22}m_{33}-m_{23}^{2}}*({\bar{v}}_{r}-\varepsilon \bar{r}) \end{array}$$

After coordinate transformation, Equation (12) shows that ${\dot{\bar{v}}}_{r}$ has no direct actuation. Hence, the USV studied in this paper is under-actuated.

### 2.3. Problem Description of Path Tracking

The planned path is an expected path formed by a number of track reference points under the geodetic coordinate system P(Ω), where Ω is parameter variable and Ω≥0. It satisfies the parameter update rate $\dot{\Omega }=\frac{U}{{\left({x^{\prime }}_{p}^{2}(\Omega )+{y^{\prime }}_{p}^{2}(\Omega )\right)}^{1/2}}>0$, where ${\bar{y^{\prime }}}_{p}(\Omega )=\frac{d{\bar{y}}_{p}(\Omega )}{d\Omega }$, ${\bar{x^{\prime }}}_{p}(\Omega )=\frac{d{\bar{x}}_{p}(\Omega )}{d\Omega }$, and U is the absolute resultant velocity of USV motion. $\gamma_{p}(\Omega )$ is the included angle between the tangent direction at any point on the parameter path $\left(x_{p}(\Omega ),y_{p}(\Omega )\right)$ and the longitudinal axis $X_{p}$ of the {i} system. The angle is positive when it is clockwise. The expression is as follows:

(18) $$\gamma_{p}(\Omega )=\arctan(\frac{y^{\prime }(\Omega )}{x^{\prime }(\Omega )}),\;\gamma_{p}(\Omega )\in (-\pi ,\pi )$$

**Figure 2:**
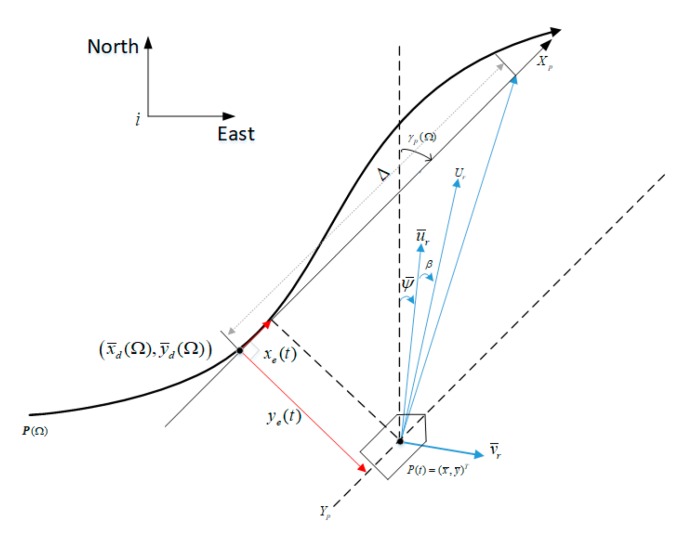
Line-of-sight guidance strategy.

The current position of USV in the {i} system is $P(t)={\left(\bar{x},\bar{y}\right)}^{T}$, and the expected velocity is ${\bar{u}}_{rd}{}_{}$. In combination with Figure 2, the longitudinal tracking error is as follows:

(19) $$\begin{array}{c} y_{e}(t)=-(\bar{x}-{\bar{x}}_{p}(\Omega ))\sin\gamma_{p}(\Omega )+\;\\ \;(\bar{y}-{\bar{y}}_{p}(\Omega ))\cos\gamma_{p}(\Omega ) \end{array}$$

Equation (19) is differentiated as follows:

(20) $$\begin{array}{lll} {\dot{y}}_{e}(t) & = & -\sin\gamma_{p}(\Omega )(\dot{\bar{x}}-{\dot{\bar{x}}}_{p}(\Omega ))\\ & & -{\dot{\gamma }}_{p}(\Omega )\cos\gamma_{p}(\Omega )(\bar{x}-{\bar{x}}_{p}(\Omega ))\\ & & \;+\cos\gamma_{p}(\Omega )(\dot{\bar{y}}-{\dot{\bar{y}}}_{p}(\Omega ))\\ & & -\;{\dot{\gamma }}_{p}(\Omega )\sin\gamma_{p}(\Omega )\;(\bar{y}-{\bar{y}}_{p}(\Omega )) \end{array}$$

From Equation (18), we obtain:

(21) $$\dot{\bar{x}}\sin(\gamma_{p}(\Omega ))-\dot{\bar{y}}\cos(\gamma_{p}(\Omega ))=0$$

Based on the geometry in Figure 1, it is learned that:

(22) $$\begin{array}{l} (\bar{x}-{\bar{x}}_{p}(\Omega ))\cos\gamma_{p}(\Omega )\;\\ -\;(\bar{y}-{\bar{y}}_{p}(\Omega ))\sin\gamma_{p}(\Omega )=0 \end{array}$$

Equations (21) and (22) are substituted into Equation (20) to obtain:

(23) $$\begin{array}{ll} {\dot{y}}_{e}(t) & =\;({\bar{u}}_{r}\sin\bar{\psi }+{\bar{v}}_{r}\cos\bar{\psi }+V_{y})\cos(\gamma_{p}(\Omega ))\\ & -({\bar{u}}_{r}\cos\bar{\psi }-{\bar{v}}_{r}\sin\bar{\psi }+V_{x})\sin(\gamma_{p}(\Omega ))\\ & =\;U\sin(\bar{\psi }+\beta -\gamma_{p}(\Omega )) \end{array}$$

where U is the resultant velocity in the {i} system, and $\beta =\arctan({\bar{v}}_{r},{\bar{u}}_{r})$ is the lateral sliding angle generated by the transverse velocity of USV and the disturbance in the external environment.

## 3. Control Objectives

The problem of multi-USV formation control could be broken down into the problem of single USV path tracking and the problem of multi-USV coordinated formation. First of all, a path tracking controller is designed for single USV to realize the control objective of spatial motion converging to the expected path. After that, the forward motion velocity of each USV is adjusted considering the USV path tracking velocity and position to realize the control objective of multi-USV coordinated formation.

### 3.1. Single USV Path Tracking Control Objective

Considering the above analysis of path and model, this paper employs the integral line-of-sight guidance subsystem and the heading control subsystem cascade. Guidance system could be used to determine the heading of under-actuated USV based on the real-time position, attitude information and planned route of under-actuated USV. Heading control subsystem could determine the expected steering gear manipulation signal based on the deviation between expected heading and actual heading of guidance system, so as to control the under-actuated USV and make its path tracking position and heading converge to the equilibrium position. Heading control subsystem controls the velocity of under-actuated USV converging to the expected velocity.

Therefore, the expected control objective in this paper could be described as follows:

(24) $$\underset{t\to \infty }{\lim}y_{e}(t)=0$$

(25) $$\underset{t\to \infty }{\lim}\bar{\psi }(t)={\bar{\psi }}_{d}$$

where ${\bar{\psi }}_{d}$ is the heading reference signal given by guidance subsystem, i.e., expected heading angle.

### 3.2. Coordinated Formation Control Objective

**Figure 3:**
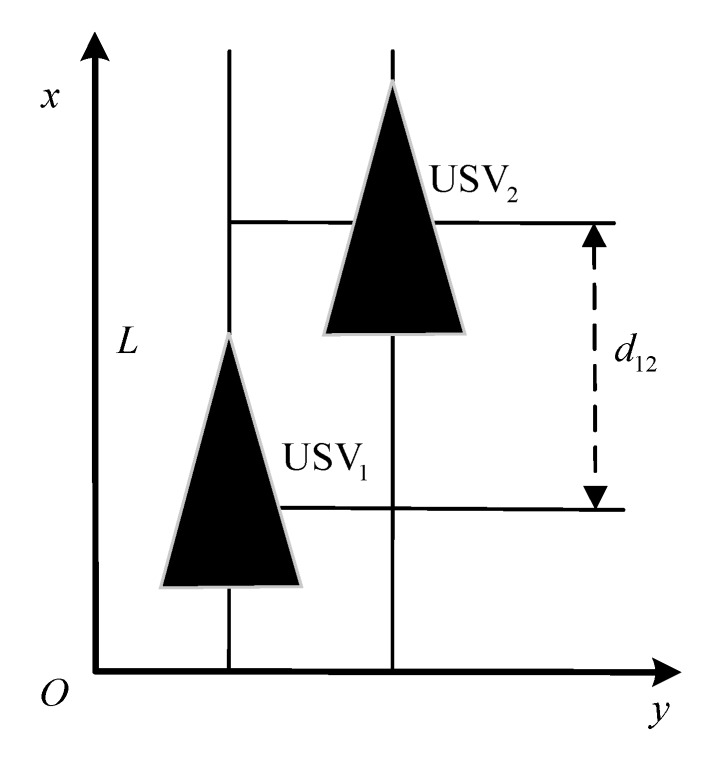
Description of USV formation motion.

As shown in Figure 3, in order to realize the USV formation coordination control, it is considered that the constant d>0 represents the expected distance between USVs. A controller is designed to ensure that the USV distance eventually converges to d. Therefore, the problem of formation system control could be described as follows:

(26) $$\lim{\bar{x}}_{j}\left(t\right)-{\bar{x}}_{i}\left(t\right)-d_{ji}=0,\;j,i=1,\ldots ,n$$

The USV forward velocity ${\bar{u}}_{r}$ converges to the expected velocity ${\bar{u}}_{d}$. In other words, longitudinal velocity error ${\bar{u}}_{e}$ converges to 0. Therefore, there is:

(27) $$\underset{t\to \infty }{\lim}{\bar{u}}_{r_{j}}\left(t\right)-{\bar{u}}_{d}\left(t\right)=0,\;j=1,\ldots ,n$$

Above all, the problem of multi-USV formation coordination path tracking could be divided into:

1. Designing a single USV path tracking controller for USV control to realize the control objective Equations (24) and (25), of position and heading, and move along the x axis at the given velocity ${\bar{u}}_{c_{j}}$;
2. Designing a controller for ${\bar{u}}_{c_{j}}$ to coordinate USVs for the expected distance d on the x axis, so as to meet the coordinated control objective Equation (26) and control the formation velocity tracking to the expected velocity ${\bar{u}}_{d}$.

## 4. Controller Design

### 4.1. Design of Path Tracking Controller Based on Adaptive Integral Line-of-Sight Guidance Algorithm

In the traditional line-of-sight (LOS) algorithm, the current position of USV is projected on the expected route and a tangent is made at the point to define the headway range on the tangent and obtain the virtual target point related to the reference point. The current position of USV and the virtual point are the LOS vectors. The resultant velocity direction of USV is guided to align with the LOS vectors for tracking. The LOS algorithm does not rely on the USV model in principle, so that it is slightly affected by the algorithm. Hence, it does not have very high requirements for parameter design. Additionally, expected heading could be calculated only using the real-time position and expected route of USV. The expected heading could be obtained and output in a real-time manner to the control system. Nevertheless, the traditional LOS algorithm may be affected by time-varying disturbance, so that its accuracy is poor during the tracking process. For this reason, integral term should be introduced to make compensation for disturbance in a real-time manner, so as to realize higher accuracy. The integral LOS (ILOS) guidance strategy under the relative velocity in the traditional algorithm could be expressed as follows:

(28) $${\bar{\psi }}_{d}=\gamma_{p}(\Omega )-\beta -\arctan(\frac{1}{\Delta }(y_{e}(t)+y_{\mathrm{int}}))$$

(29) $${\dot{y}}_{\mathrm{int}}=\gamma \frac{\Delta Uy_{e}(t)}{\sqrt{\Delta^{2}+{\left(y_{e}(t)+\Delta y_{\mathrm{int}}\right)}^{2}}},\;\gamma >0$$

The adaptive headway range Δ is designed to improve the ILOS and define it as the function of longitudinal tracking error:

(30) $$\Delta =\Delta_{\min}+\Delta_{\chi }e^{-\sigma \left(y_{e}(t)\right)}{}^{{}^{1/2}}$$

where σ is the constant parameter that is strictly greater than 0 [7]; $\Delta_{\min}$ is the minimum headway range, and $\Delta_{\chi }=\Delta_{\max}-\Delta_{\min}$ is the maximum increment of headway range. When USV deviates far away from the planned route, the tracking error $y_{e}$ is high, and $\underset{y_{e}(t)\to \infty }{\lim}e^{-\sigma \left(y_{e}(t)\right)}{}^{{}^{1/2}}\approx 0$. At this time, headway range is $\Delta =\Delta_{\min}$, so that USV approaches the given expected route fast. When USV gets close to the planned route, the tracking error $y_{e}$ is low, and $\underset{y_{e}(t)\to 0}{\lim}e^{-\sigma \left(y_{e}(t)\right)}{}^{{}^{1/2}}\approx 1$. At this time, headway range is $\Delta =\Delta_{\max}$, so that USV could keep the stable tracking on the given track.

### 4.2. Design of Heading and Velocity Controller

In this section, the feedback linearization design control law is followed to make the USV heading and velocity converge to the expected values.

Based on the expected heading ${\bar{\psi }}_{d}$ and expected angular velocity ${\dot{\bar{\psi }}}_{d}$ given in the guidance subsystem [35], Equation (11) is used to obtain the heading control law as follows:

(31) $$T_{r}=\frac{m_{22}m_{33}-m_{23}^{2}}{b_{32}m_{22}-b_{32}m_{23}}\left(\begin{array}{l} -F_{r}({\bar{u}}_{r},{\bar{v}}_{r},\bar{r})+{\ddot{\bar{\psi }}}_{d}-\\ k_{1}(\dot{\bar{\psi }}-{\dot{\bar{\psi }}}_{d})-k_{2}(\bar{\psi }-{\bar{\psi }}_{d}) \end{array}\right)$$

where $k_{1}$ and $k_{2}$ are the constants that are strictly greater than 0.

Equation (13) is used to design the velocity control law as follows:

(32) $$T_{u}=\frac{m_{11}}{b_{11}}\left(\begin{array}{c} -F_{u_{r}}({\bar{u}}_{r},{\bar{v}}_{r},\bar{r})+\frac{b_{11}}{m_{11}}{\bar{u}}_{rd}+\\ \;{\dot{\bar{u}}}_{rd}-k_{3}({\bar{u}}_{r}-{\bar{u}}_{rd}) \end{array}\right)$$

here $k_{3}$ is a constant that is greater than 0, which ensures that velocity exponent converges to the expected value.

### 4.3. Design of Coordinated Formation Controller

The influence of seawater flow on the design of path tracking controller has been taken into account. Therefore, $V_{x}$ is ignored in the design of coordination controller. Equation (8) is transformed to obtain:

(33) $$\begin{array}{lll} \dot{\bar{x}} & = & {\bar{u}}_{r}-{\bar{u}}_{r}\left(1-\cos\bar{\psi }\right)-{\bar{v}}_{r}\sin\bar{\psi }\\ & = & {\bar{u}}_{c}+{\bar{u}}_{e}-{\bar{u}}_{r}\frac{\left(1-\cos\bar{\psi }\right)}{\bar{\psi }}\bar{\psi }-{\bar{v}}_{r}\frac{\sin\bar{\psi }}{\bar{\psi }}\bar{\psi } \end{array}$$

where ${\bar{u}}_{c}$ is velocity reference command, ${\bar{u}}_{e}={\bar{u}}_{r}-{\bar{u}}_{c}$.

Because of $\bar{\psi }={\bar{\psi }}_{d}+{\bar{\psi }}_{e}=\frac{-\tan^{-1}\left(y_{e}/\Delta \right)}{y_{e}}y_{e}+{\bar{\psi }}_{e}$, it is substituted into Equation (33) to obtain:

(34) $$\dot{\bar{x}}={\bar{u}}_{c}+f\left(y_{e},\bar{\psi },\bar{u},\bar{v}\right)\varpi$$

where $f\left(\cdot \right)={\left[\begin{array}{c} \frac{{\bar{u}}_{r}\left(1-\cos\bar{\psi }\right)-{\bar{v}}_{r}\sin\bar{v}}{\bar{\psi }}\cdot \frac{\tan^{-1}\left(y_{e}/\Delta \right)}{y_{e}}\\ \frac{{\bar{v}}_{r}\sin\bar{\psi }-{\bar{u}}_{r}\left(1-\cos\bar{\psi }\right)}{\bar{\psi }} \end{array}\right]}^{T}$ and $\varpi ={\left[{\bar{u}}_{e},y_{e},{\bar{\psi }}_{e}\right]}^{T}$. According to the trigonometric function, $\sin x/x,\left(1-\cos x\right)/x,\tan^{-1}\left(x\right)/x$ is globally bounded. Moreover, the exponent of the element in the vector ϖ converges to 0 under the path tracking controller designed in the above section. Hence, the velocity $\bar{u}\left(t\right)$ on the direction of the x axis asymptotically converges to the reference command velocity ${\bar{u}}_{c}\left(t\right)$.

By then, the controller ${\bar{u}}_{c_{j}},j=1,\ldots ,n$ is designed to make USV formation realize the coordination control objective (27), and satisfy the reference command velocity. Assuming that there is h>0, the expected velocity ${\bar{u}}_{d}\left(t\right)$ satisfies:

(35) $${\bar{u}}_{d}\left(t\right)\in \left[u_{\min}+h,u_{\max}-h\right],\;\forall t\geq t_{0}$$

where $u_{\max}>u_{\min}>0$.

The controller is designed as follows:

(36) $${\bar{u}}_{c_{j}}={\bar{u}}_{d}\left(t\right)-\Gamma \left(\sum_{i=1}^{n}\left({\bar{x}}_{j}-{\bar{x}}_{i}-d_{ji}\right)\right),j=1,\ldots ,n$$

where $d_{ji}={\bar{x}}_{d_{j}}-{\bar{x}}_{d_{i}}$ represents the distance between the jth USV and the ith USV in parallel with the x axis. Γ(·) is the bounded continuous differentiable increasing function and satisfies $\Gamma^{\prime }\left(0\right)>0$, Γ(0)=0 and Γ(x)∈(−h,h).

## 5. Stability Verification

### 5.1. Stability of Guidance System

During the process of USV motion, β varies with time under the change of heading angle $\bar{\psi }$ and transverse velocity ${\bar{v}}_{r}$. Compared with the motion of USV, such change is very slow. The following assumptions could be given:

**Assumption 1.**β is a constant and varies very slightly, i.e., $\dot{\beta }=0$.

Therefore, Equation (23) could be written as:

(37) $${\dot{y}}_{e}(t)=U\sin(\bar{\psi }-\gamma_{p}(\Omega ))\;+\;U\cos(\bar{\psi }-\gamma_{p}(\Omega ))\beta$$

After defining $\tilde{\psi }=\bar{\psi }-\psi_{d}$ as the heading angle error of USV, Equation (37) could be rewritten as:

(38) $$\begin{array}{c} {\dot{y}}_{e}(t)=U\sin(\tilde{\psi }+\psi_{d}-\gamma_{p}(\Omega ))+\\ \;U\cos(\tilde{\psi }+\psi_{d}-\gamma_{p}(\Omega ))\beta \end{array}$$

Equation (38) is expanded to obtain:

(39) $$\begin{array}{c} {\dot{y}}_{e}(t)=U\sin(\tilde{\psi }+\psi_{d}-\gamma_{p}(\theta ))+\\ \;U\cos(\tilde{\psi }+\psi_{d}-\gamma_{p}(\theta ))\beta \end{array}$$

Moreover, there is:

(40) $$\sin\left(\tan^{-1}\left(-\frac{1}{\Delta }y_{e}(t)-y_{\mathrm{int}}\right)\right)=\frac{y_{e}(t)+\Delta y_{\mathrm{int}}}{\sqrt{\Delta^{2}+{\left(y_{e}(t)+\Delta y_{\mathrm{int}}\right)}^{2}}}$$

(41) $$\cos\left(\tan^{-1}\left(-\frac{1}{\Delta }y_{e}(t)+y_{\mathrm{int}}\right)\right)=\frac{\Delta }{\sqrt{\Delta^{2}+{\left(y_{e}(t)+\Delta y_{\mathrm{int}}\right)}^{2}}}$$

Equations (40) and (41) are substituted into Equation (38) to obtain:

(42) $$\begin{array}{c} {\dot{y}}_{e}(t)=-\frac{U(y_{e}(t)+\Delta y_{\mathrm{int}})}{\sqrt{\Delta^{2}+{\left(y_{e}(t)+\Delta y_{\mathrm{int}}\right)}^{2}}}\;+\\ \;\frac{U\Delta }{\sqrt{\Delta^{2}+{\left(y_{e}(t)+\Delta y_{\mathrm{int}}\right)}^{2}}}\beta +\\ \;U[\phi_{1}(y_{e}(t),\tilde{\psi })+\beta \phi_{2}(y_{e}(t),\tilde{\psi })]\tilde{\psi } \end{array}$$

where

(43) $$\phi_{1}(y_{e}(t),\tilde{\psi })=\frac{\sin\tilde{\psi }}{\tilde{\psi }}\cos(\psi_{d}-\gamma_{p}(\Omega ))+\frac{\cos\tilde{\psi }-1}{\tilde{\psi }}\sin(\psi_{d}-\gamma_{p}(\Omega ))$$

(44) $$\phi_{2}(y_{e}(t),\tilde{\psi })=-\frac{\sin\tilde{\psi }}{\tilde{\psi }}\sin(\psi_{d}-\gamma_{p}(\theta ))+\frac{\cos\tilde{\psi }-1}{\tilde{\psi }}\cos(\psi_{d}-\gamma_{p}(\theta ))$$

Obviously, there is $\left|\sin\tilde{\psi }/\tilde{\psi }\right|\leq 1$, $\left|(\cos\tilde{\psi }-1)/\tilde{\psi }\right|\leq 0.73$, $\frac{\Delta }{\sqrt{\Delta^{2}+(y_{e}+y_{\mathrm{int}}{)}^{2}}}\leq 1$, and $\frac{y_{e}+y_{\mathrm{int}}}{\sqrt{\Delta^{2}+(y_{e}+y_{\mathrm{int}}{)}^{2}}}\leq 1$. Thus, $\phi_{1}(y_{e}(t),\tilde{\psi })$ and $\phi_{2}(y_{e}(t),\tilde{\psi })$ are bounded and satisfy:

(45) $$\left|\phi_{1}(y_{e}(t),\tilde{\psi })\right|\leq 1.73,\;\left|\phi_{2}(y_{e}(t),\tilde{\psi })\right|\leq 1.73$$

Let $\hat{\beta }$ be the estimation of drift angle β from adaptive observer, and the estimation error is $\tilde{\beta }=\beta -\hat{\beta }$. When the accurate tracking of heading and velocity is realized, the virtual control input could be $y_{\mathrm{int}}=\hat{\beta }$ and meets Assumption 3. Thus, Equation (42) may be rewritten as:

(46) $${\dot{y}}_{e}(t)=-\frac{U(y_{e}(t)+\Delta \hat{\beta })}{\sqrt{\Delta^{2}+{\left(y_{e}(t)+\Delta \hat{\beta }\right)}^{2}}}\;+\frac{U\Delta }{\sqrt{\Delta^{2}+{\left(y_{e}(t)+\Delta \hat{\beta }\right)}^{2}}}\beta$$

In other words, there is:

(47) $${\dot{y}}_{e}(t)=-\frac{Uy_{e}(t)}{\sqrt{\Delta^{2}+{\left(y_{e}(t)+\Delta \hat{\beta }\right)}^{2}}}\;+\frac{U\Delta }{\sqrt{\Delta^{2}+{\left(y_{e}(t)+\Delta \hat{\beta }\right)}^{2}}}\tilde{\beta }$$

The Lyapunov function $V_{1}$ is designed and defined as follows:

(48) $$V_{1}(y_{e}(t),t,\tilde{\beta })=\frac{1}{2}y_{e}{\left(t\right)}^{2}+\frac{1}{2k}{\tilde{\beta }}^{2}>0$$

where $y_{e}(t)\neq 0$, $\tilde{\beta }\neq 0$ and k>0.

It is differentiated to obtain:

(49) $${\dot{V}}_{1}(y_{e}(t),t,\tilde{\beta })=y_{e}(t){\dot{y}}_{e}(t)+\frac{1}{k}\tilde{\beta }\dot{\tilde{\beta }}=-\frac{Uy_{e}{\left(t\right)}^{2}}{\sqrt{\Delta^{2}+{\left(y_{e}(t)+\Delta \hat{\beta }\right)}^{2}}}+\left(\frac{U\Delta y_{e}(t)}{\sqrt{\Delta^{2}+{\left(y_{e}(t)+\Delta \hat{\beta }\right)}^{2}}}+\frac{1}{k}\dot{\tilde{\beta }}\right)$$

As a result of $\dot{\tilde{\beta }}=-\dot{\hat{\beta }}$, Equation (29) is substituted into Equation (49) to obtain:

(50) $${\dot{V}}_{1}(y_{e}(t),t,\tilde{\beta })\;=-\frac{Uy_{e}{\left(t\right)}^{2}}{\sqrt{\Delta^{2}+{\left(y_{e}(t)+\Delta \hat{\beta }\right)}^{2}}}\;\leq 0$$

Therefore, guidance subsystem has uniform global asymptotic stability (UGAS) at the equilibrium point $y_{e}(t)=0$.

Additionally, there is μ>0. When $y_{e}(t)$ satisfies $D=\left\{y_{e}(t)\in R\;|\;\left|y_{e}(t)\right|\leq \mu \right\}$, ${\dot{V}}_{1}(y_{e}(t),t)$ satisfies:

(51) $${\dot{V}}_{1}(y_{e}(t),t,\tilde{\beta })\;=\frac{-Uy_{e}{\left(t\right)}^{2}}{\sqrt{\Delta^{2}+{\left(y_{e}(t)+\Delta \hat{\beta }\right)}^{2}}}\;\leq -wy_{e}{\left(t\right)}^{2}$$

where $0<w<\frac{U}{\sqrt{\Delta^{2}+{\left(y_{e}(t)+\Delta \hat{\beta }\right)}^{2}}}$. Therefore, guidance subsystem has uniform local exponential stability (ULES) at the equilibrium point $y_{e}(t)=0$. UGAS and ULES are equivalent to the global K-exponential stability (GKES), and their stability is conceptually stronger than UGAS but weaker than global exponential stability (GES).

### 5.2. Stability of Heading and Velocity Controller

The formula for dynamic tracking error of heading and velocity control subsystem is as follows:

(52) $$\left[\begin{array}{c} \dot{\tilde{\psi }}\\ \ddot{\tilde{\psi }}\\ {\dot{\tilde{u}}}_{r} \end{array}\right]=\left[\begin{array}{ccc} \begin{array}{c} 0\\ -k_{2}\\ 0 \end{array} & \begin{array}{c} 1\\ -k_{1}\\ 0 \end{array} & \begin{array}{c} 0\\ 0\\ -k_{3} \end{array} \end{array}\right]\left[\begin{array}{c} \tilde{\psi }\\ \dot{\tilde{\psi }}\\ {\tilde{u}}_{r} \end{array}\right]$$

where $\tilde{\psi }=\bar{\psi }-{\bar{\psi }}_{d}$, $\dot{\tilde{\psi }}=\dot{\bar{\psi }}-{\dot{\bar{\psi }}}_{d}$, and ${\tilde{u}}_{r}={\bar{u}}_{r}-{\bar{u}}_{rd}$ are the tracking errors of heading angle, heading angular velocity, and velocity, respectively.

When the system is linear and time-invariant, and $k_{1}$, $k_{2}$, and $k_{3}$ in the coefficient matrix are all greater than 0, the real parts of all characteristic roots in the system are negative. The coefficient matrix of the system is Hurwitz matrix. Hence, the equilibrium state of the system $\left({\tilde{u}}_{r},\tilde{\psi },\;\tilde{r})=(0,0,0\right)$ is global exponential stable [35,36], i.e., the control objectives $\underset{t\to \infty }{\lim}\bar{\psi }(t)={\bar{\psi }}_{d}$, $\underset{t\to \infty }{\lim}\bar{u}\left(t\right)-{\bar{u}}_{d}\left(t\right)=0$ are achieved.

### 5.3. Stability of Path Tracking System

The position error dynamic equation and heading and velocity error dynamic equation are written in the following cascade:

(53) $$\begin{array}{l} \sum 1\;:\;{\dot{y}}_{e}(t)=-\frac{U(y_{e}(t)+\Delta y_{\mathrm{int}})}{\sqrt{\Delta^{2}+{\left(y_{e}(t)+\Delta y_{\mathrm{int}}\right)}^{2}}}\;+\\ \;\frac{U\Delta \beta }{\sqrt{\Delta^{2}+{\left(y_{e}(t)+\Delta y_{\mathrm{int}}\right)}^{2}}}\;+g(t,y_{e},\xi )\xi \end{array}$$

(54) $$\sum 2:\;\dot{\xi }=\left[\begin{array}{c} \dot{\tilde{\psi }}\\ \ddot{\tilde{\psi }}\\ {\dot{\tilde{u}}}_{r} \end{array}\right]=\left[\begin{array}{ccc} \begin{array}{c} 0\\ -k_{2}\\ 0 \end{array} & \begin{array}{c} 1\\ -k_{1}\\ 0 \end{array} & \begin{array}{c} 0\\ 0\\ -k_{3} \end{array} \end{array}\right]\left[\begin{array}{c} \tilde{\psi }\\ \dot{\tilde{\psi }}\\ {\tilde{u}}_{r} \end{array}\right]$$

where $\xi =\left[\begin{array}{c} \tilde{\psi }\\ \dot{\tilde{\psi }}\\ {\tilde{u}}_{r} \end{array}\right]$, $g(t,y_{e},\xi )={\left[\begin{array}{c} U_{r}\phi (y_{e},\tilde{\psi })\\ 0\\ 0 \end{array}\right]}^{T}$.

When the system ∑1 has uniform semi-global exponential stability and the system ∑2 has uniform local exponential stability, the closed-loop control system ∑1−∑2 has uniform global asymptotic and uniform semi-global exponential stability at the position $\left(y_{e},{\tilde{u}}_{r},\tilde{\psi },\dot{\tilde{\psi }})=(0,0,0,0\right)$.

Verification: As a result of $|\phi (y_{e},\tilde{\psi })|\leq 1.73$, the link term $g(t,y_{e},\xi )$ of the system ∑1−∑2 satisfies:

(55) $$\left\|g(t,y_{e},\xi )\right\|\leq 1.73U_{r}$$

Since the systems ∑1 and ∑2 have been proved to have uniform semi-global exponential stability and uniform local exponential stability in the above section, the system ∑1−∑2 has uniform semi-global exponential stability at the position $\left(y_{e},{\tilde{u}}_{r},\tilde{\psi },\dot{\tilde{\psi }})=(0,0,0,0\right)$. This fulfils a control objective $\underset{t\to \infty }{\lim}y_{e}(t)=0$, $\underset{t\to \infty }{\lim}\bar{\psi }(t)={\bar{\psi }}_{d}$, i.e., USV could realize the stable tracking of expected path under the control of path tracking algorithm.

### 5.4. Stability of Coordinated Formation Controller

Equation (36) is substituted into Equation (34) to obtain:

(56) $${\dot{\bar{x}}}_{j}={\bar{u}}_{d}\left(t\right)-\Gamma \left(\sum_{i}^{n}\left({\bar{x}}_{j}-{\bar{x}}_{i}-d_{ji}\right)\right)+z_{j}\varpi_{j},\;j=1,\ldots ,n$$

Let ${\tilde{x}}_{j}={\bar{x}}_{j}-{\bar{x}}_{d_{j}},j=1,\ldots ,n$, so that Equation (56) could be written as:

(57) $${\tilde{x}}_{j}=-\Gamma \left(\sum_{i=1}^{n}\left({\tilde{x}}_{j}-{\tilde{x}}_{i}\right)\right)+z_{j}\varpi_{j},\;j=1,\ldots ,n$$

Equation (57) is transformed in the form of vector $\tilde{x}={\left[{\tilde{x}}_{1},\ldots .{\tilde{x}}_{n}\right]}^{T}$, and $\Gamma \left(\tilde{x}\right)=\left[g\left({\tilde{x}}_{1}\right),\ldots ,g\left({\tilde{x}}_{n}\right)\right]$. Thus, Equation (57) could be rewritten as:

(58) $$\tilde{x}=-\Gamma \left(L\tilde{x}\right)+Z\omega$$

where $\omega ={\left[\varpi_{1}^{T},\ldots ,\varpi_{n}^{T}\right]}^{T}$, Z is block diagonal matrix. The diagonal element is $z_{j},j=1,\ldots ,n$. Since $z_{j}$ is bounded, Z is bounded. The matrix L is the Laplacian matrix of communication topological graph G, so that $L=\left\{l_{ji}\right\}\in R^{n\times n}$. In the meanwhile, there is:

(59) $$l_{ji}\left\{ \begin{array}{l} \delta_{j},j=1\\ -1,j\neq 1,\mathrm{and}\left(j,i\right)\in E,j,i=1,\ldots ,n\\ 0,\;\mathrm{other} \end{array}\right.$$

where $\delta_{j}$ is the out-degree of the vertex j. As shown in Equation (59), $Lv_{1}=0$, $v_{1}={\left[1,\ldots ,1\right]}^{T}$ and $v_{1}\in R^{n}$. According to the graph theory, matrix L has a zero eigen-value, if and only if communication topographic graph G contains a globally reachable point.

Stability analysis is conducted as follows:

**Theorem** **1.** *Considering the kinematic and kinetic model of USV Equation (1), it is assumed that* $u_{d}(t)$ *is continuously differentiable, and satisfies Equation (35);* $u_{cj}(t)$ *satisfies* $u_{cj}(t)\in (u_{\min},u_{\max}),\;j=1,\ldots ,n$ *; and the communication topographic graph G between USVs has at least one globally reachable point. In the USV formation, the initial position of each USV is random. Under the effect of controller Equations (30), (31), and (32), USVs move at the expected velocity obtained from Equation (36). Eventually, all exponents could converge to the expected path and the expected formation is realized. In other words, control objective Equations (24) and (25) are fulfilled.*

**Theorem** **2.** *Considering that Theorem 1 is justified, and the system in Equation (58) is under the coordination transformation* $\mu =T\;\tilde{x}\;,T\in R^{\left(n-1\right)\times n}$ *, the following formulas are established:*

*(1)* ${\tilde{x}}_{1}=\ldots ={\tilde{x}}_{n}$ *, i.e.,* μ=0

*(2)* $\dot{\mu }\;=g\left(\mu \right)+G\left(\omega ,u_{c}\right)\omega$ *, where* $G\left(\omega ,u_{c}\right)$ *is globally bounded.*

*(3) There is positive definite and radially unbounded Lyapunov function* V=V(μ) *, which satisfies:*

(60) $$\frac{\partial V}{\partial \mu }\left(\mu \right)g\left(\mu \right)\leq W\left(\mu \right)<0,\forall \mu \in R^{n-1}$$

(61) $$\left\|\frac{\partial V}{\partial \mu }\left(\mu \right)\right\|\leq C_{1},\forall \mu \in R^{n-1}$$

*The system* $\dot{\mu }=g\left(\mu \right)$ *has global asymptotic stability and uniform local exponential stability.*

**Verification:** It is assumed that the communication topographic graph G in Equation (58) contains s globally reachable points, and 1≤s≤n. The matrix L could be written as:

(62) $$L=\left[\begin{array}{cc} L_{1} & L_{2}\\ 0 & L_{3} \end{array}\right]$$

where $L_{3}\in R^{s\times s}$ is Laplacian matrix, and its element is globally reachable. Hence, $G\left(L_{3}\right)$ is strongly connected. $L_{1}\in R^{\left(n-s\right)\times \left(n-s\right)}$ is semi-Hurwitz, and satisfies:

(63) $$PL_{1}+L_{1}^{T}P=Q,Q=Q^{T}>0$$

where P is positive definite and a diagonal matrix.

$L_{3}$ is semi positive definite, and contains a zero eigen-value. The zero eigen-value corresponds to the eigenvector $1_{s}=\left[1,\;\ldots ,1\right]\in R^{s}$. Thus $L_{3}$ could be divided into $L_{3}=M_{3}M_{3}^{T}$ and $M_{3}\in R^{s\times \left(s-1\right)}$. Therefore, $\mu =T\tilde{x}$ could be rewritten as:

(64) $$\mu =\left[\begin{array}{ll} L_{1} & L_{2}\\ 0 & M_{3}^{T} \end{array}\right]\tilde{x}=T\tilde{x}$$

**Verification 1.** There is μ=0, so that $L\mu =\left[\begin{array}{ll} I & 0\\ 0 & M_{3} \end{array}\right]=0$, i.e., $\mu =\alpha 1_{n},\alpha \in R$. Therefore, ${\tilde{x}}_{j}={\tilde{x}}_{i},j,i=1,\ldots ,n$.

**Verification 2.** Equations (58), (62), and (64), are combined as follows:

(65) $$\begin{array}{ll} \dot{\mu } & =\left[\begin{array}{c} -L_{1}\Gamma_{1}(\mu_{1})-L_{2}\Gamma_{2}(M_{3}\mu_{2})\\ -M_{3}^{T}\Gamma_{2}\left(M_{3}\mu_{2}\right) \end{array}\right]+TZ\omega \\ & =g\left(\mu \right)+G\omega \end{array}$$

where $\mu ={\left[\mu_{1}^{T},\mu_{2}^{T}\right]}^{T}$, $\mu_{1}\in R^{\left(s-r\right)}$, $\mu_{2}\in R^{r}$, G=TZ, T is constant matrix, and Z is globally bounded, so that G is globally bounded.

**Verification 3.** Equation (65) is used to obtain:

(66) $$\mu =g\left(\mu \right)=\left[\begin{array}{c} {\dot{\mu }}_{1}\\ {\dot{\mu }}_{2} \end{array}\right]=\left[\begin{array}{l} -L\Gamma \left(\mu_{1}\right)-L_{2}\Gamma \left(M_{3}\mu_{2}\right)\\ -M_{3}^{T}\Gamma_{2}\left(M_{3}\mu_{2}\right) \end{array}\right]$$

Considering the Lyapunov function:

(67) $$\begin{array}{ll} V & =\frac{\lambda }{2}{\left\|\mu_{2}\right\|}^{2}+\sum_{i=1}^{n}\int_{0}^{\mu_{1}}p_{i}\Gamma \left(y\right)dy\\ & =\frac{\lambda }{2}{\left\|\mu_{2}\right\|}^{2}+\sum_{i=1}^{n}\int_{0}^{\mu_{1}}P\Gamma_{1}\left(y\right)dy \end{array}$$

where $p_{i}$ is the diagonal element of the positive definite diagonal matrix P. Meanwhile, we select λ>0 since Γ∈[0,ℓ],ℓ>0. V is radially unbounded, so that there is:

(68) $$\dot{V}=-\lambda {\left(M_{3}\mu_{2}\right)}^{T}\Gamma_{2}\left(M_{3}\mu_{2}\right)-\frac{1}{2}\Gamma_{1}^{T}\left(\mu_{1}\right)\left[PL_{1}+L_{1}^{T}P\right]\Gamma_{1}\left(\mu_{1}\right)-\Gamma_{1}^{T}\left(\mu_{1}\right)PL_{2}\Gamma_{2}\left(M_{3}\mu_{2}\right)$$

Let $\kappa =M_{3}\mu_{2}$. Equation (68) could be simplified to:

(69) $$\dot{V}\leq -\lambda \kappa^{T}\Gamma_{2}\left(\kappa \right)-\frac{q_{m}}{2}{\left\|\Gamma_{1}^{T}\left(\mu_{1}\right)\right\|}^{2}+c\left\|\Gamma_{2}\left(\mu_{1}\right)\right\|\cdot \left\|\Gamma_{2}\left(\kappa \right)\right\|$$

where $q_{m}$ is the minimum Eigen-value of the positive definite matrix Q in Equation (63), and it is positive, $c\geq \left\|PL_{2}\right\|>0$. Since Γ∈[0,ℓ],ℓ>0, there is $0<\Gamma^{\prime }\left(x\right)<\ell$ and ∀x∈R. Hence, $\frac{x}{\Gamma \left(x\right)}\geq \frac{1}{\ell }$, ∀x∈R. Then Equation (69) could be rewritten as:

(70) $$\dot{V}\leq -\frac{\lambda }{\ell }{\left\|\Gamma_{2}\left(\kappa \right)\right\|}^{2}-\frac{q_{m}}{2}{\left\|\Gamma_{1}^{T}\left(\mu_{1}\right)\right\|}^{2}+c\left\|\Gamma_{1}\left(\mu_{1}\right)\right\|\cdot \left\|\Gamma_{2}\left(\kappa \right)\right\|$$

We select $\lambda \geq \ell \left({\left[\frac{c}{\sqrt{2q_{m}}}\right]}^{2}+\alpha \right)$ and α>0, so that there is:

(71) $$\begin{array}{ll} \dot{V} & \leq -{\left(\frac{c}{\sqrt{2q_{m}}}\left\|\Gamma_{2}\left(\kappa \right)\right\|-\sqrt{\frac{q_{m}}{2}}\left\|\Gamma_{1}\left(\Gamma_{1}\left(\mu_{1}\right)\right)\right\|\right)}^{2}-\alpha {\left\|\Gamma_{2}\left(\kappa \right)\right\|}^{2}\\ & =-W\left(\left(\Gamma_{1}\left(\mu_{1}\right),\Gamma_{2}\left(\kappa \right)\right)\right) \end{array}$$

where $W=W\left(\Gamma_{1}\left(\mu_{1}\right),\Gamma_{2}\left(\kappa \right)\right)$ is the positive definite function of $\Gamma_{1}\left(\mu_{1}\right)$ and $\Gamma_{2}\left(\kappa \right)$. Γ(x)=0 if and only if x=0. The matrix $M_{3}$ is a full-rank matrix. If and only if $\mu_{1}=\mu_{2}=0$, W=0. In other words, time-invariant system Equation (58) has uniform global asymptotic stability at the position μ=0.

After linearization, Equation (66) is changed to:

(72) $$\Delta \dot{\mu }=-\Gamma^{\prime }(0)\left[\begin{array}{ll} L_{1} & L_{2}M_{3}\\ 0 & M_{3}^{T}M_{3} \end{array}\right]\Delta \mu$$

where $-L_{1}$ is a Hurwitz matrix, $-M_{3}^{T}M_{3}$ is a Hurwitz matrix as well. Hence, the system Equation (72) has uniform global asymptotic stability at the position μ=0. Hence, the system Equation (66) has uniform global exponential stability at the position μ=0.

Let $\tilde{V}=\ln\left(V+1\right)$, $\tilde{V}$ is calculated to obtain:

(73) $$\dot{\tilde{V}}\leq -\frac{1}{V\left(\mu \right)+1}W\left(\Gamma_{1}\left(\mu_{1}\right),\Gamma_{2}\left(\kappa \right)\right)=-\tilde{W}\left(\mu \right)<0$$

Meanwhile,

(74) $$\begin{array}{ll} \left\|\frac{\partial \tilde{V}}{\partial \mu }\right\| & \leq \frac{1}{V+1}\left(\gamma \left\|\mu_{2}\right\|+\left\|\Gamma_{1}\left(\mu_{1}\right)\right\|\cdot \left\|P\right\|\right)\\ & \leq \gamma \frac{\left\|\mu_{2}\right\|}{V+1}+\left\|\Gamma_{1}\left(\mu_{2}\right)\right\|\cdot \left\|P\right\|\\ & \leq \gamma \frac{\left\|\mu_{2}\right\|}{\frac{\gamma }{2}{\left\|\mu_{2}\right\|}^{2}+1}+\left\|\Gamma_{1}\left(\mu_{1}\right)\right\|\cdot \left\|P\right\|\\ & \leq C_{1} \end{array}$$

where $C_{1}>0$, and $\left\|\Gamma_{1}\left(\mu_{1}\right)\right\|$ is globally bounded, Equation (61) is verified. In other words, Theorem 2 is verified.

Above all, USV formation system could tend to be stable under the control of the controller Equation (36). Hence, Theorem 1 is verified. The verification of its stability fulfils a control objective $\lim{\bar{x}}_{j}\left(t\right)-{\bar{x}}_{i}\left(t\right)-d_{ji}=0,j,i=1,\ldots ,n$, i.e., USV formation could converge to the expected formation and be maintained under the effect of formation coordination controller.

The verification ends.

## 6. Simulation and Experiment

To verify the effectiveness of the proposed control approach in this paper, the USV model in Reference [36] is employed to perform the simulation experiment for single USV and formulation path tracking. It is assumed that each USV in the simulation satisfies the following model parameters of USV, as shown in Table 1:

### 6.1. Single USV Simulation Experiment

**Figure 4:**
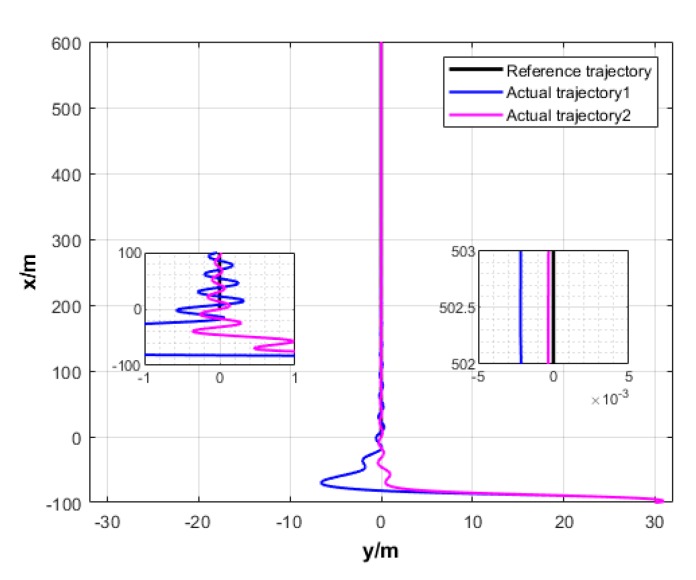
Straight track of USV under two strategies.

**Figure 5:**
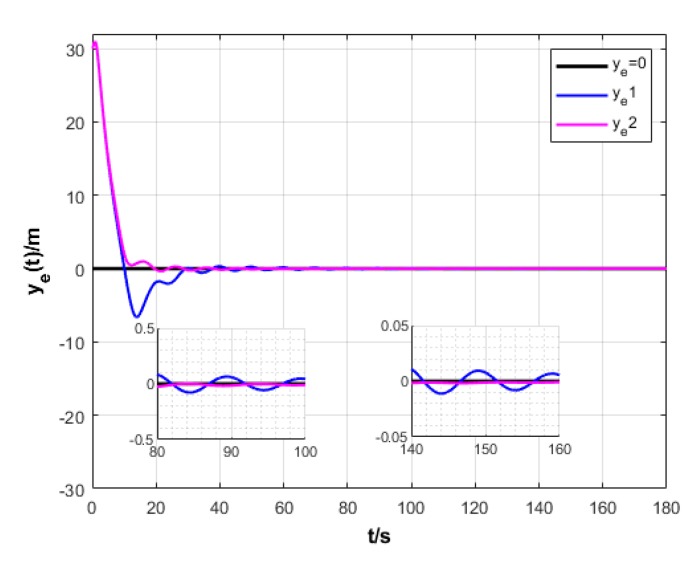
Transverse error of USV under two strategies.

**Figure 6:**
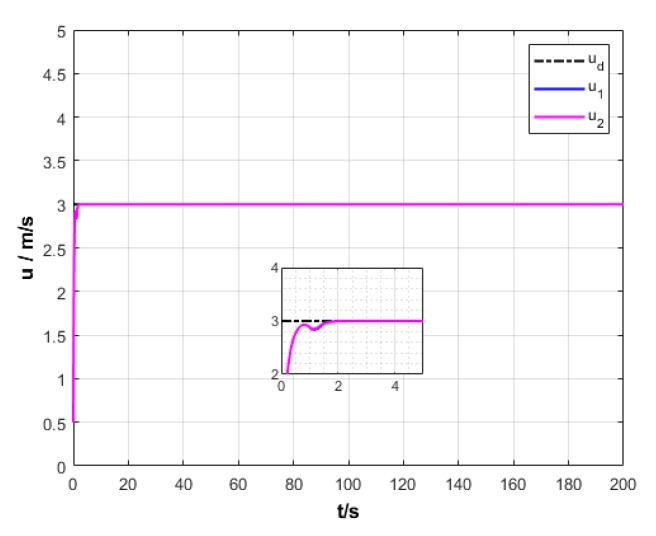
Longitudinal velocity of USV under two strategies.

**Figure 7:**
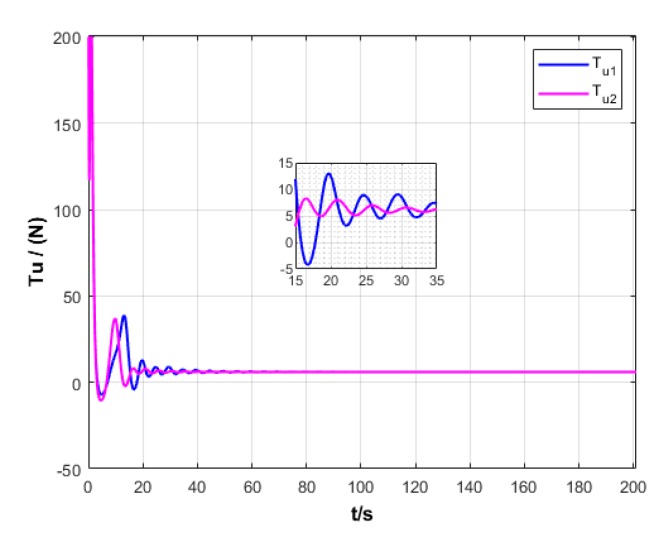
Forward thrust of USV under two strategies.

**Figure 8:**
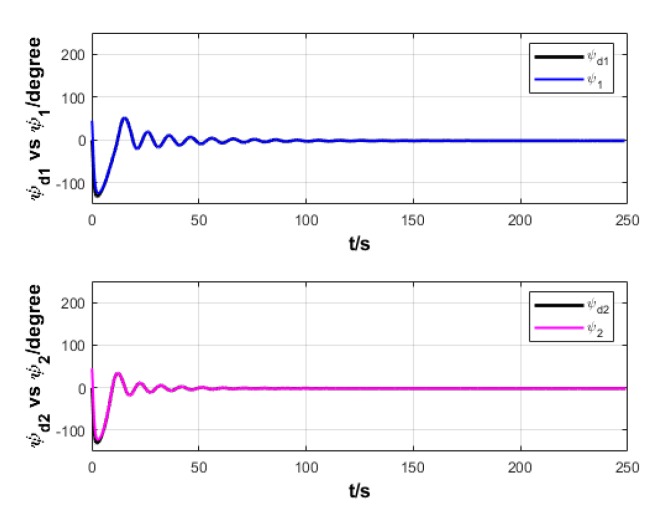
Heading angle of USV under two strategies.

**Figure 9:**
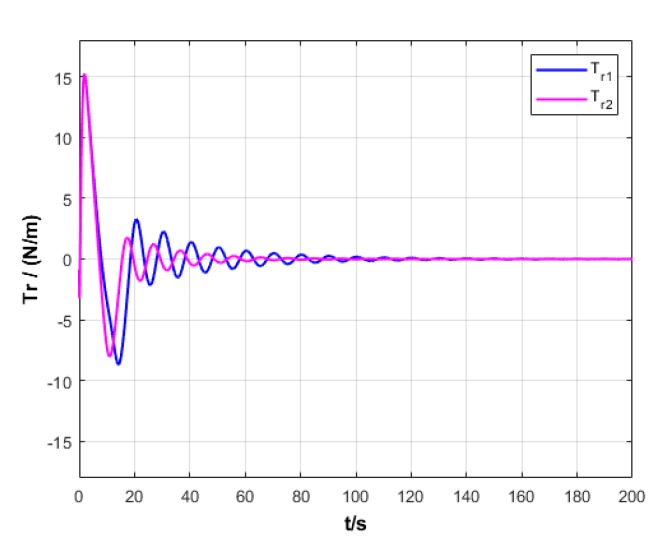
Turning torque of USV under two strategies.

**Figure 10:**
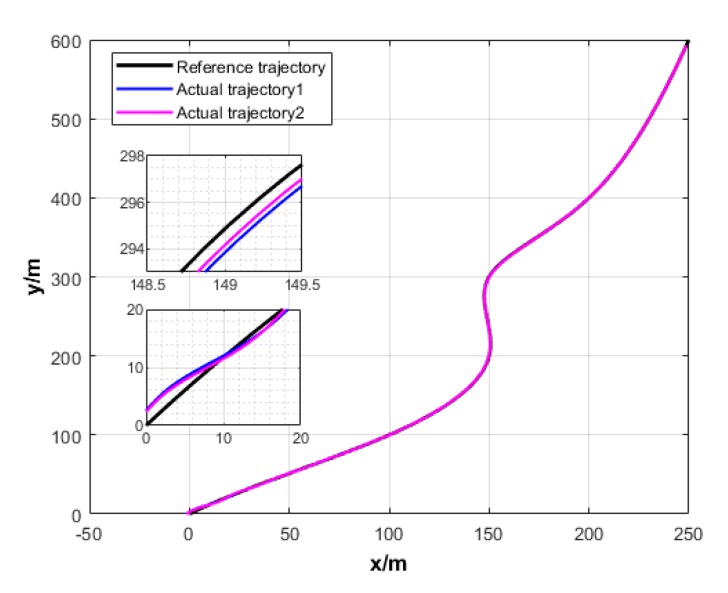
Curved path tracking of USV under two strategies.

**Figure 11:**
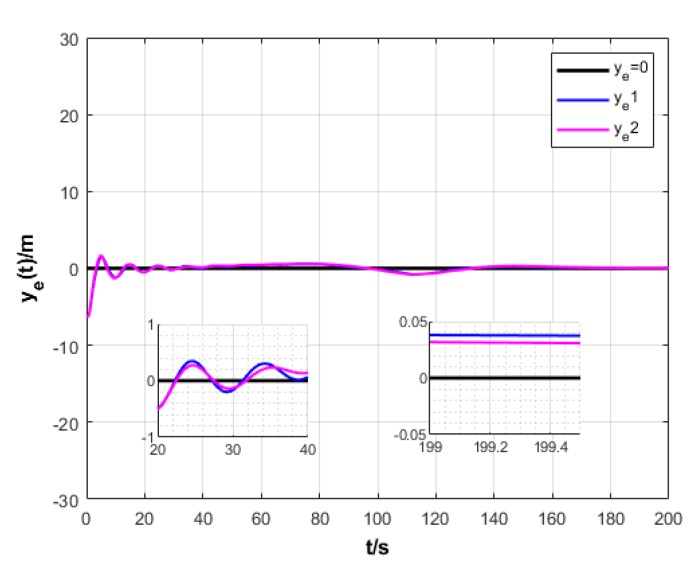
Transverse error variation of USV under two strategies.

**Figure 12:**
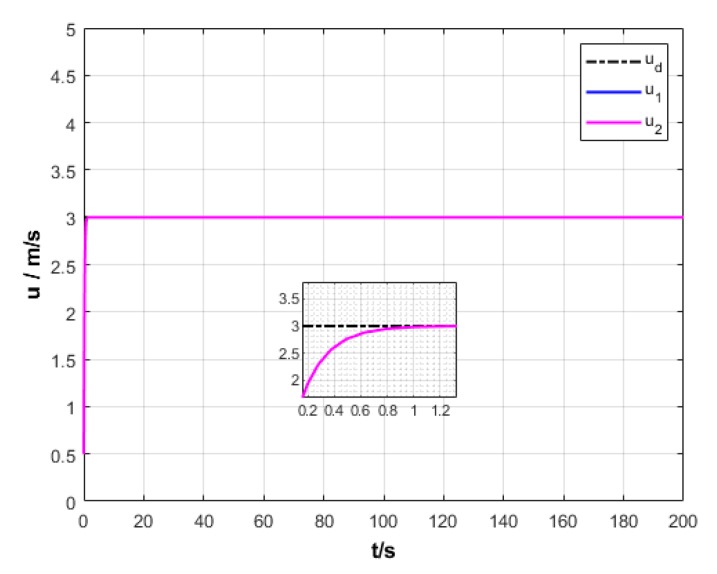
Relative longitudinal velocity variation of USV under two strategies.

**Figure 13:**
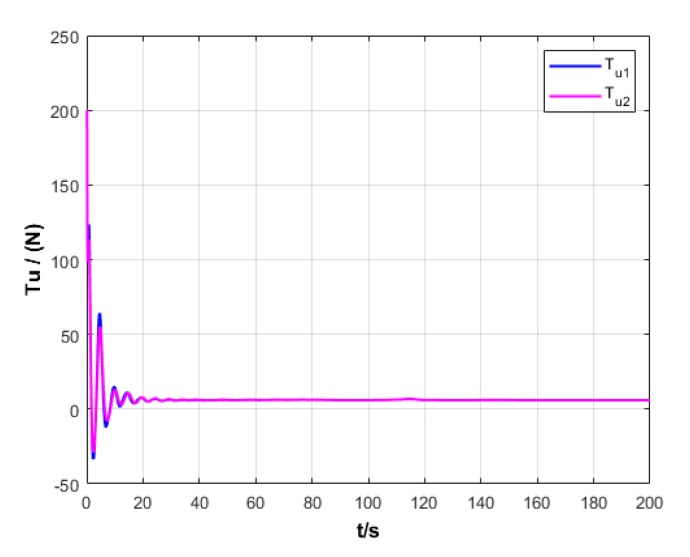
Forward thrust variation of USV under two strategies.

**Figure 14:**
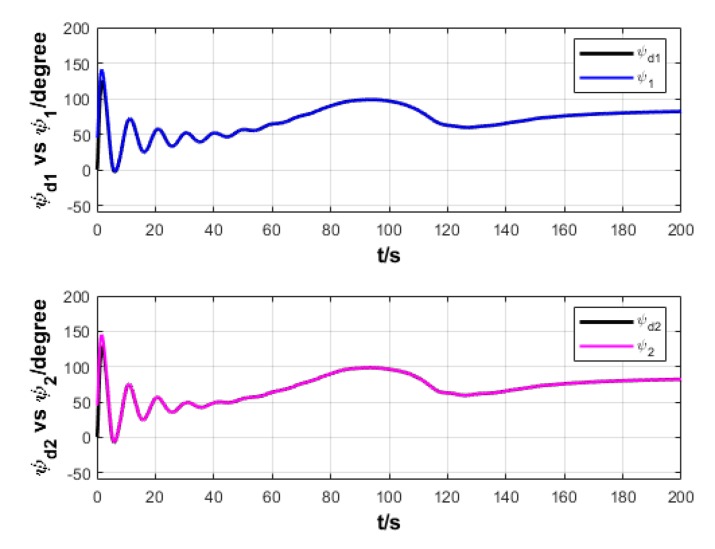
Heading angle variation of USV under two strategies.

**Figure 15:**
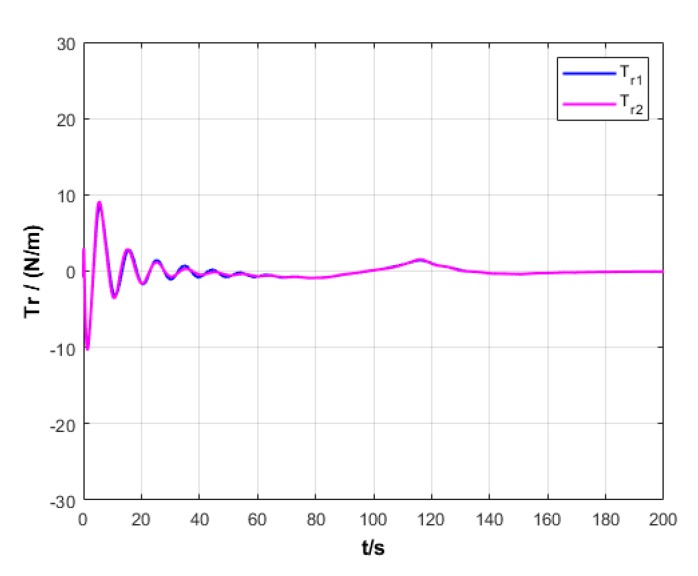
Turning torque variation of USV under two strategies.

A USV path is straight and curved. In this section, simulation experiment is conducted using the path tracking algorithm with the traditional LOS guidance strategy and the control algorithm with the proposed ILOS guidance strategy in this paper to compare straight and curved paths. Among them, the parameters for heading and velocity subsystem are $k_{1}=5$, $k_{2}=3$, and $k_{3}=5$. The parameters for guidance system are selected as follows: under the traditional LOS strategy, there is γ=0.005 and Δ=8 m; under the proposed strategy in this paper, there is γ=0.0009, δ=3, $\Delta_{\min}=6$ m, and $\Delta_{\chi }=8$ m. The simulation is presented in Figure 4, Figure 5, Figure 6, Figure 7, Figure 8, Figure 9, Figure 10, Figure 11, Figure 12, Figure 13, Figure 14 and Figure 15. Among them, the control algorithm with the traditional guidance strategy is represented by the subscript 1, and abbreviated as Algorithm 1, while the control algorithm with the proposed strategy is indicated by the superscript 2, and abbreviated as Algorithm 2.

(1) Straight path

The expected route of USV is y=0, and the expected velocity is $u_{d}=3\;m/s$. The initial position of USV is set to (30,−100). The initial heading is $\psi_{0}=\pi /4\;\mathrm{rad}$, and the initial velocity is $u_{r}=0.5\;m/s$. The flow rate of seawater is set to $U_{c}=0.2\;m/s$, and the flow direction is $\psi_{c}=30^{\circ }$.

(2) Curved path

The expected track is a continuous curve generated through approximation by five polynomials, that is, (0,0), (100,100), (150,200), (150,300), (200,400), and (250,600). The expected velocity is $u_{d}=3\;m/s$. The initial position of USV is (−5,−5), and the initial velocity is $u_{r}=0.5\;m/s$. The heading is $\psi_{0}=\pi /4\;\mathrm{rad}$. The flow rate of seawater is set to $U_{c}=0.2\;m/s$, and the flow direction is $\psi_{c}=30^{\circ }$.

As revealed in Figure 4 and Figure 10, Algorithms 1 and 2 could both guarantee the rapid convergence of USV to the straight and curved paths. Compared with Algorithm 1, Algorithm 2 has faster convergence. At the position with the largest curvature variation of the curved path, Algorithm 2 has lower oscillation and overshoot than Algorithm 1.

As shown in Figure 5 and Figure 11, two algorithms could guarantee that the longitudinal position error of USV $y_{e}$ converges to approximately 0. However, Algorithm 2 has shorter oscillation and lower amplitude than Algorithm 1.

After comprehensively analyzing Figure 6, Figure 7, Figure 12, and Figure 13, two algorithms could guarantee the velocity stability of USV approximately $u_{d}=3\;m/s$ in the tracking of both straight and curved paths, but Algorithm 1 has larger oscillation amplitude of the forward thrust in the early section of the track than Algorithm 2. It means that Algorithm 2 has lower initial thrust than Algorithm 1. For this reason, Algorithm 2 could control USV to achieve the expected velocity at the lower expense of fuel.

After comprehensively analyzing Figure 8, Figure 9, Figure 14, and Figure 15, two algorithms could guarantee the satisfying tracking of expected heading angle, but Algorithm 2 gives the expected heading angle in a relatively smoother way and with shorter oscillation, so that the turning torque varies relatively more smoothly. Therefore, Algorithm 2 could control USV to better turn to and maintain the expected heading.

### 6.2. Formation Cruising Simulation

To verify the validity and advancement of the USV formation control algorithm proposed in this part (hereinafter referred to as Algorithm 3), a formation of 5 under-actuated USVs is employed for control. Each USV has the same model parameters and single USV path tracking simulation parameters. The software MATLAB is used for comparative simulation experiment. The control group is taken from Reference [37]. In the reference, time-variant line-of-sight guidance strategy was employed to design the path tracking controller, but it did not include model parameter perturbation and external disturbance (hereinafter referred to as Algorithm 4).

**Figure 16:**
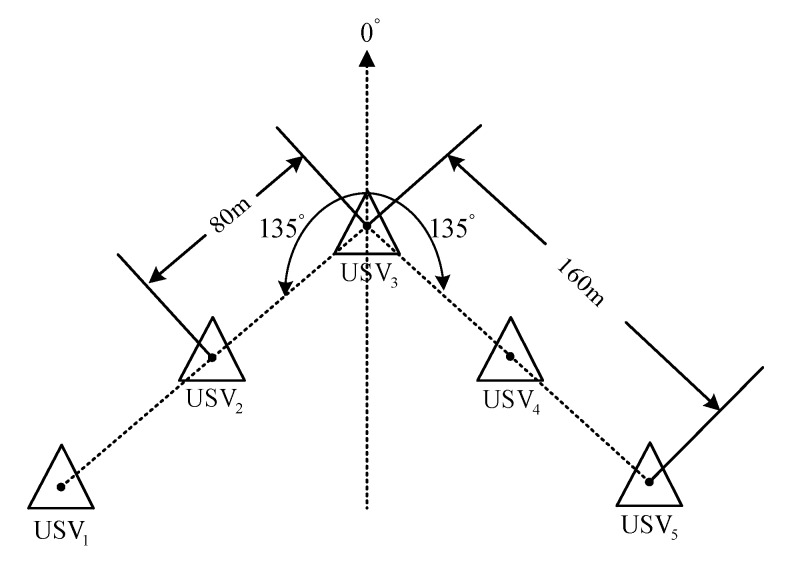
Schematic diagram of formation.

**Figure 17:**
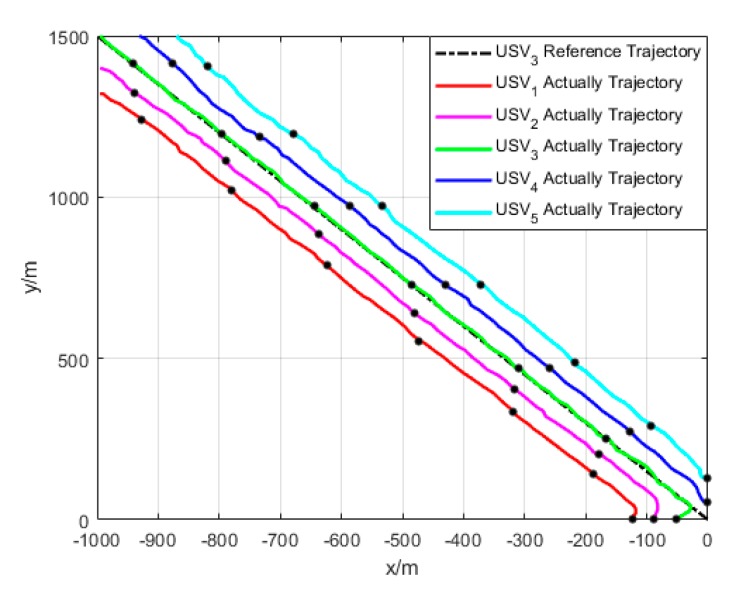
Formation trajectory (Algorithm 3).

**Figure 18:**
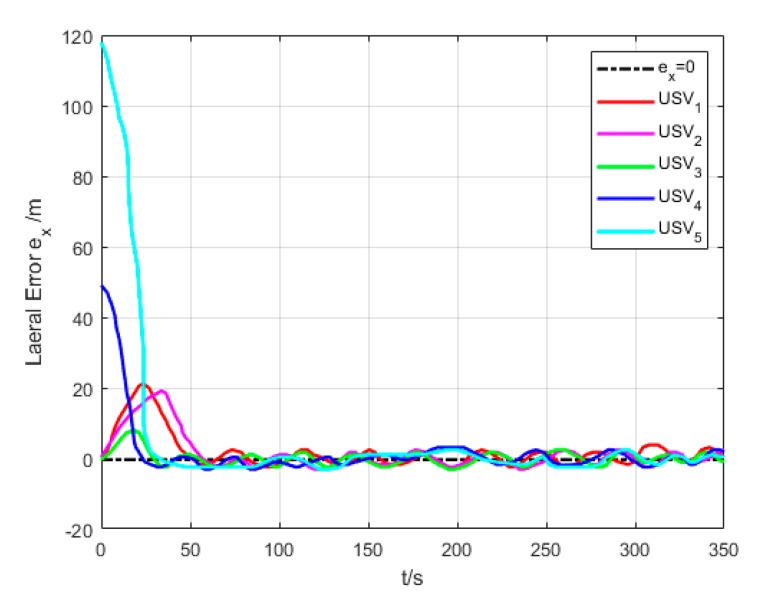
Lateral error (Algorithm 3).

The parameters of simulation environment are selected as follows: the parameters of heading and velocity subsystems are $k_{1}=5$, $k_{2}=3$, $k_{3}=5$; the parameters of guidance system are γ=0.0005, δ=3, $\Delta_{\min}=5\;m$, $\Delta_{\chi }=7\;m$. The time-variant disturbance is set to $U_{c}=0.5\;m/s$; the direction is set to $\psi_{c}(t)=1+0.2\sin(0.12)+\cos(0.3t+\frac{1}{4}\pi )$; and Γ(·) is selected as $\Gamma \left(x\right)=2/\pi \tan^{-1}(x)\in [-1,1]$. The expected path of ${\mathrm{USV}}_{1}$ is y=−x, and its expected velocity is $u_{d}=10\;kn$. The formation of USV_1_, ${\mathrm{USV}}_{2}$, ${\mathrm{USV}}_{3}$, ${\mathrm{USV}}_{4}$, and ${\mathrm{USV}}_{5}$ is shown in Figure 16:

A triangular formation is made by 5 USVs. Among them, ${\mathrm{USV}}_{3}$ is at the vertex of the triangle; ${\mathrm{USV}}_{2}$ and ${\mathrm{USV}}_{4}$ are on the right and left back of ${\mathrm{USV}}_{3}$ respectively with the angle of 135° and the distance of 80 m; ${\mathrm{USV}}_{1}$ and ${\mathrm{USV}}_{5}$ are on the right and left back of ${\mathrm{USV}}_{3}$, respectively with the angle of 135° and the distance of 160 m. The initial state of each USV is shown in Table 2:

The simulation results are given in Figure 17 and Figure 18.

As shown in Figure 17, under the effect of single USV path tracking controller, each USV enters its expected path from their respective initial state. Under the effect of formation coordination controller, each USV constantly adjusts the path to maintain their formation and overcome the influence of environmental factors. Therefore, their paths are continuously oscillating, but eventually converge to the expected path and maintain the formation.

**Figure 19:**
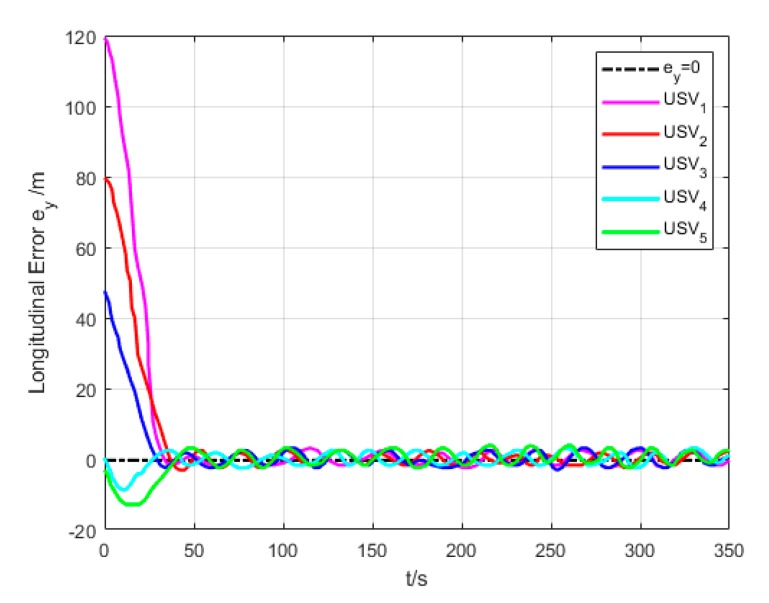
Longitudinal error (Algorithm 3).

Figure 18 and Figure 19 show the transverse and longitudinal error variations between the actual and expected positions of each USV. Due to different initial states of USVs, the errors are larger while they are making the formation. However, the transverse and longitudinal errors gradually decrease under the effect of path tracking controller and coordination controller in the middle and late section of the path. At last, their errors converge to approximately 0.

**Figure 20:**
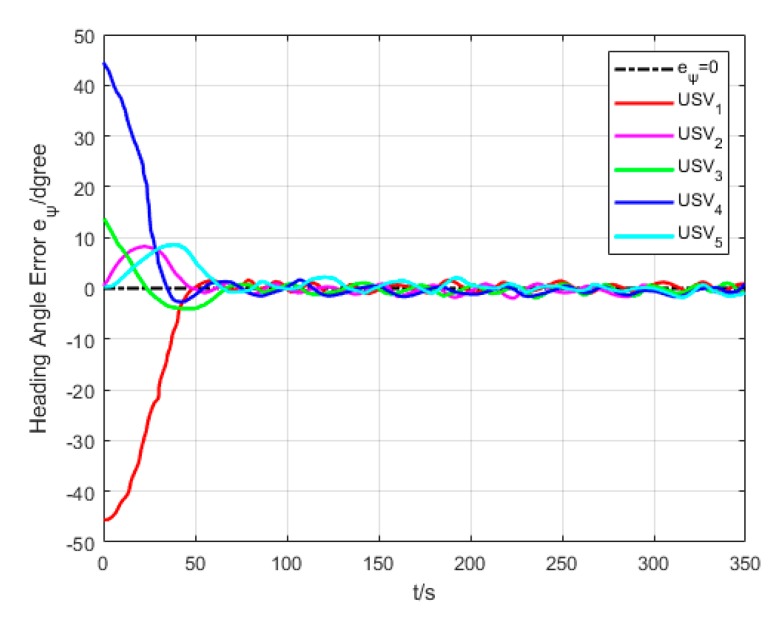
Heading angle error (Algorithm 3).

As revealed in Figure 20, each USV has a different initial heading angle, so that the oscillation amplitude of heading angle error is large at the beginning of the formation path. The heading angle error of each USV gradually converges and decreases in the middle and late section of the path.

**Figure 21:**
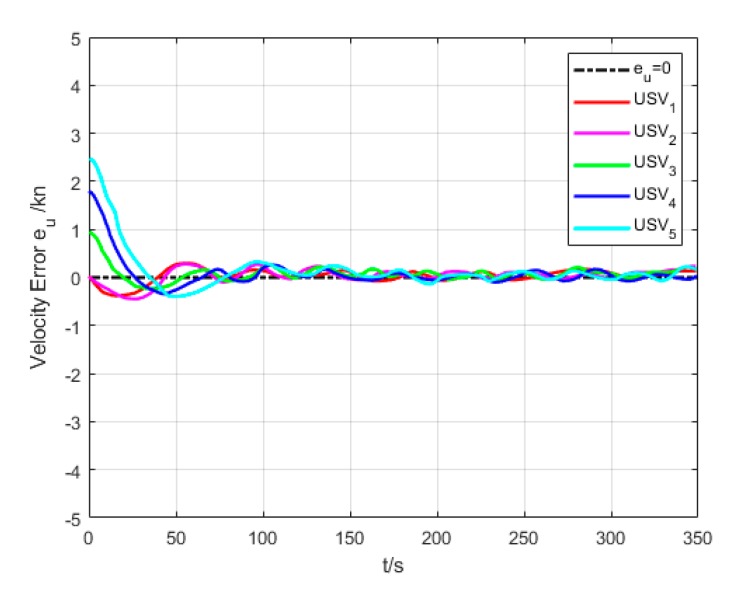
Velocity error (Algorithm 3).

As shown in Figure 21, the oscillation amplitude of velocity error is large since USVs have different initial positions and velocities while they are making the formation. While maintaining the formation, the velocity error of USVs is low, and eventually converges to approximately 0.

**Figure 22:**
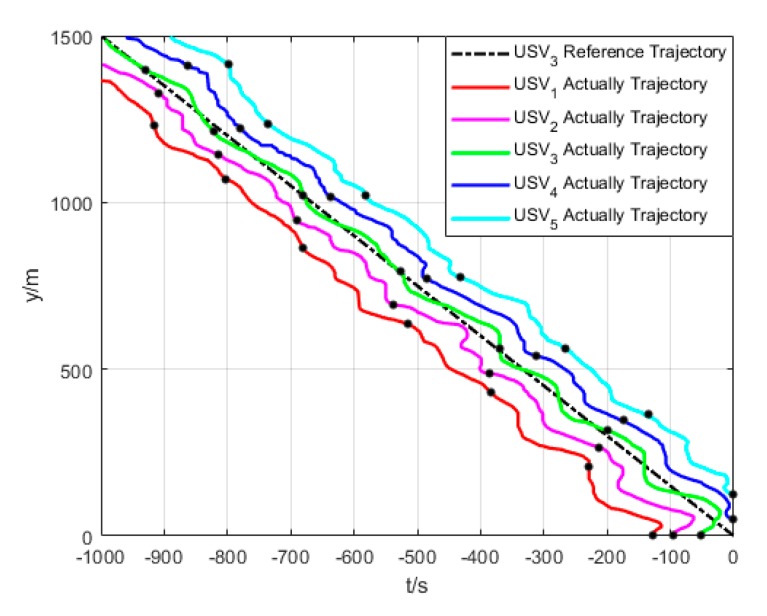
Formation trajectory (Algorithm 4).

**Figure 23:**
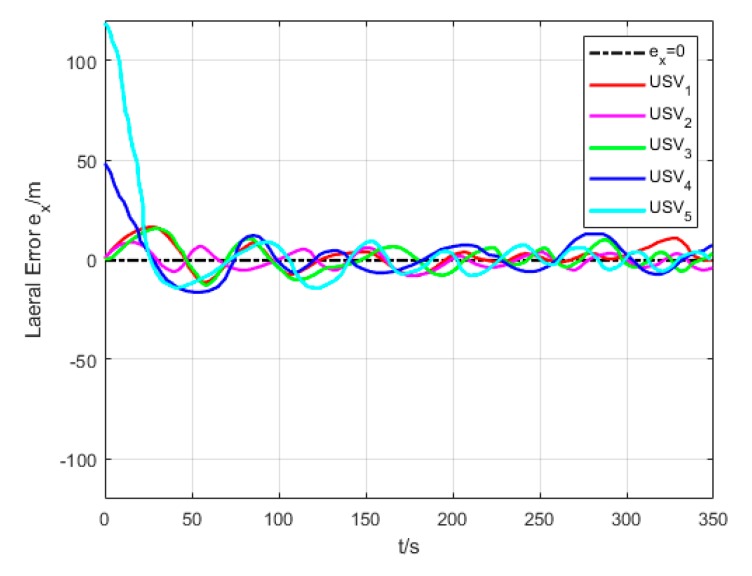
Lateral error (Algorithm 4).

**Figure 24:**
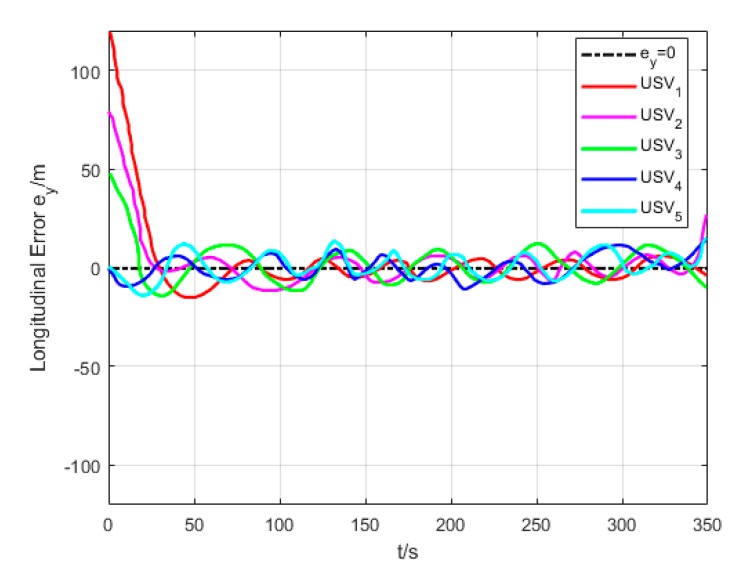
Longitudinal error (Algorithm 4).

**Figure 25:**
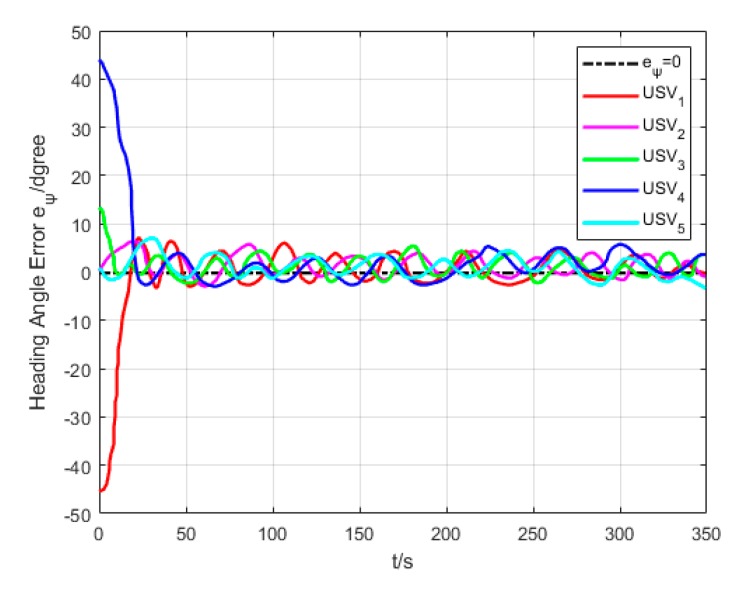
Heading angle error (Algorithm 4).

**Figure 26:**
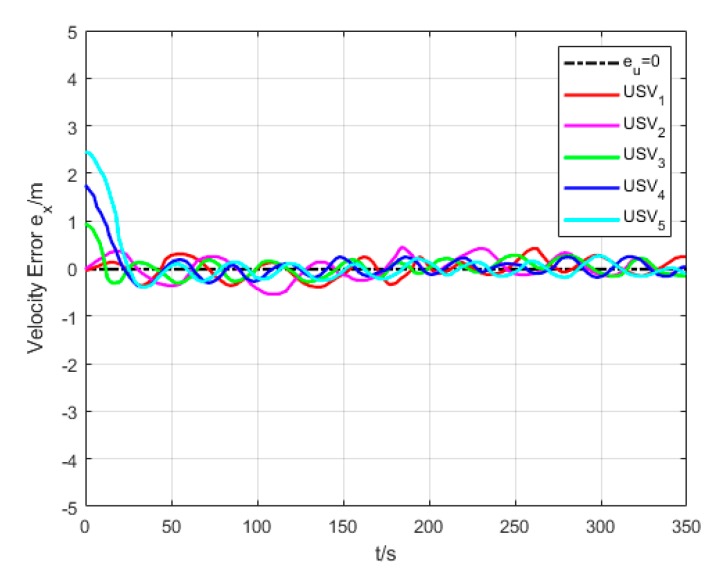
Velocity error (Algorithm 4).

As shown in Figure 22, Figure 23, Figure 24, Figure 25 and Figure 26, the formation control algorithm for the control group does not take into account the influence of model parameter perturbation and external disturbance. As a result of the set time-variant disturbance, formation control is not achieved satisfactorily. Lateral, longitudinal, heading angle, and velocity errors are all large. The expected formation under straight line condition could not be maintained.

### 6.3. USV Formation Cruising Field Experiment

**Figure 27:**
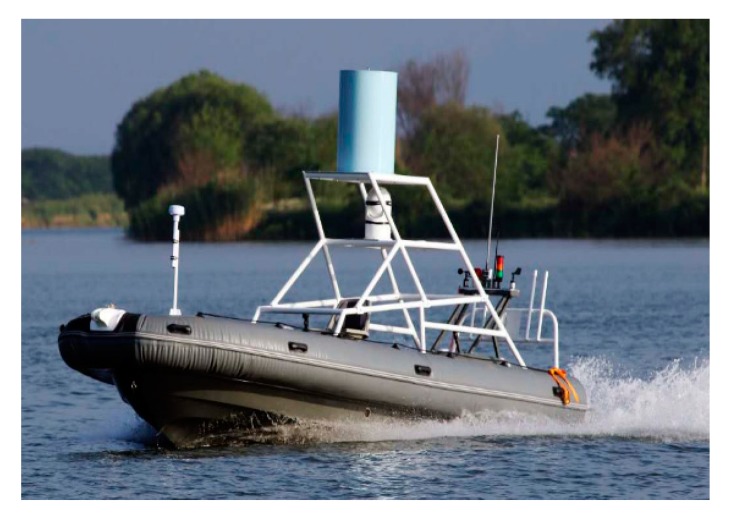
Jellyfish.

**Figure 28:**
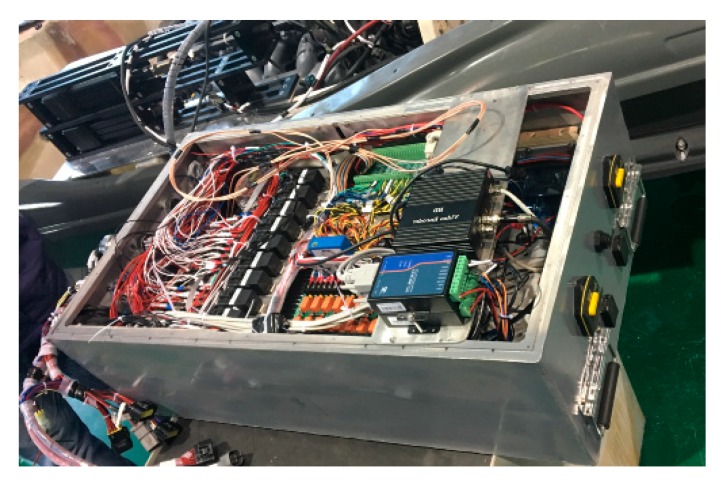
Hardware composition of USV cruising control system.

**Figure 29:**
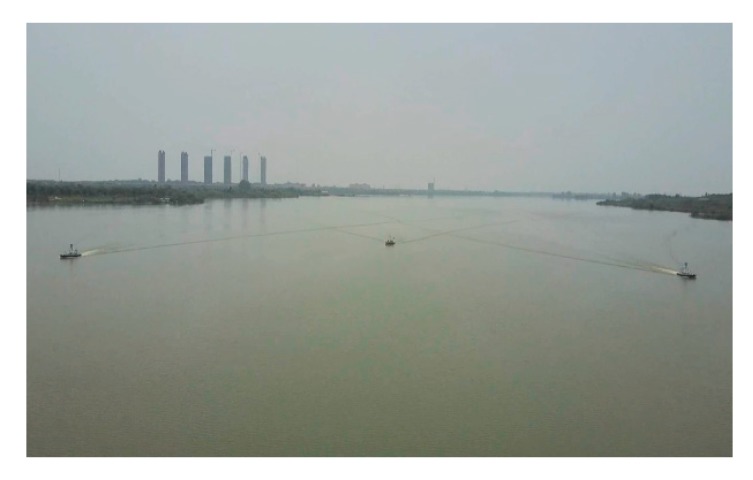
Jellyfish formation in cruising.

To verify the engineering effectiveness of the proposed USV formation control algorithm, three ‘Jellyfish’ USVs (As shown in Figure 27, Figure 28 and Figure 29) developed by the research group were used in a USV formation cruising control test in a sea area adjacent to Guzhenkou Bay in Qingtao.

**Figure 30:**
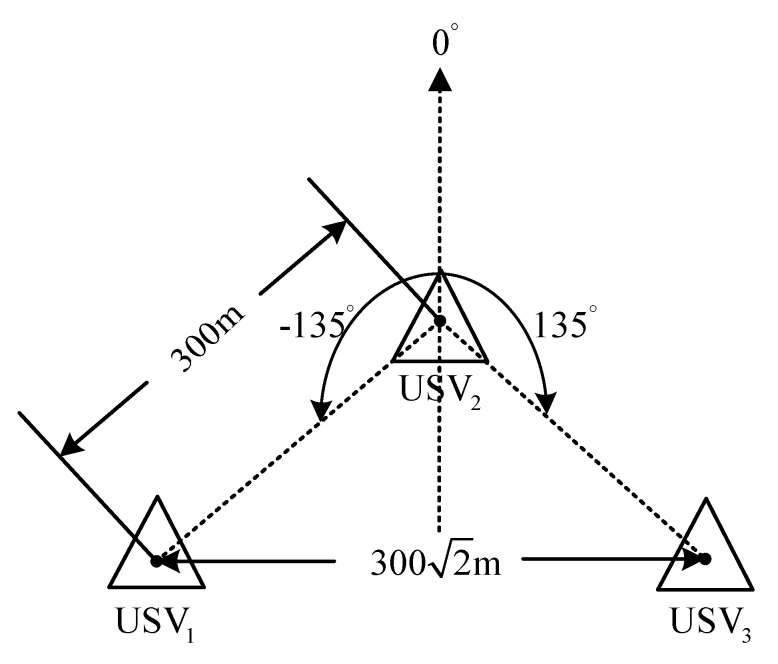
Schematic diagram of the expected formation for the USV.

**Figure 31:**
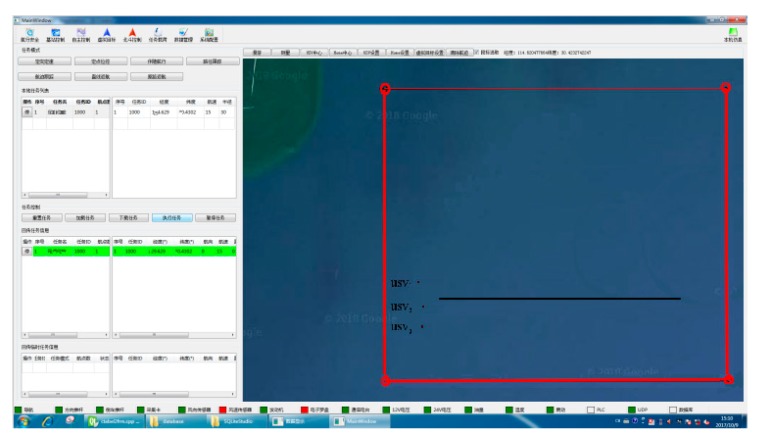
Setting interface for the expected path of ${\mathrm{USV}}_{1}$ on basestation.

The expected relative distance and direction among ${\mathrm{USV}}_{1}$, ${\mathrm{USV}}_{2}$, and ${\mathrm{USV}}_{3}$ are $l_{1,2}^{}=300\;m$, $l_{1,3}^{}=300\;m$, $\varphi_{1,2}=-135{}^{\circ}$, and $\varphi_{1,3}^{}=135{}^{\circ}$. In this case, the expected formation is as shown in Figure 30:

Figure 31 shows the setting interface for the expected path of ${\mathrm{USV}}_{1}$ on the base station with the coordinates $\left(120.150320^{\circ }E,35.7072377^{\circ }N\right)$ for the starting point and $\left(120.2173775^{\circ }E,35.7072468^{\circ }N\right)$ for the finishing point. In the figure, a red rectangle indicates the electronic security fence in service, while a black line marks the expected path of ${\mathrm{USV}}_{1}$. The expected velocity is set to $u_{d}=15\;\mathrm{kn}$. The initial parameters of each USV are shown in Table 3.

The test environment is sunny, temperature 5–12°, freeze southeast (approx. 3.7 m/s), calm sea surface, Level 0–1 sea condition, and wave height approx. 0.6 m.

During the field test, the parameters of heading and velocity subsystems are $k_{1}=6$, $k_{2}=4$, $k_{3}=3$. The parameters of guidance system are γ=0.0054, δ=2, $\Delta_{\min}=15\;m$, $\Delta_{\chi }=8\;m$. Γ(·) is $\Gamma \left(x\right)=2/\pi \tan^{-1}(x)\in [-1,1]$.

**Figure 32:**
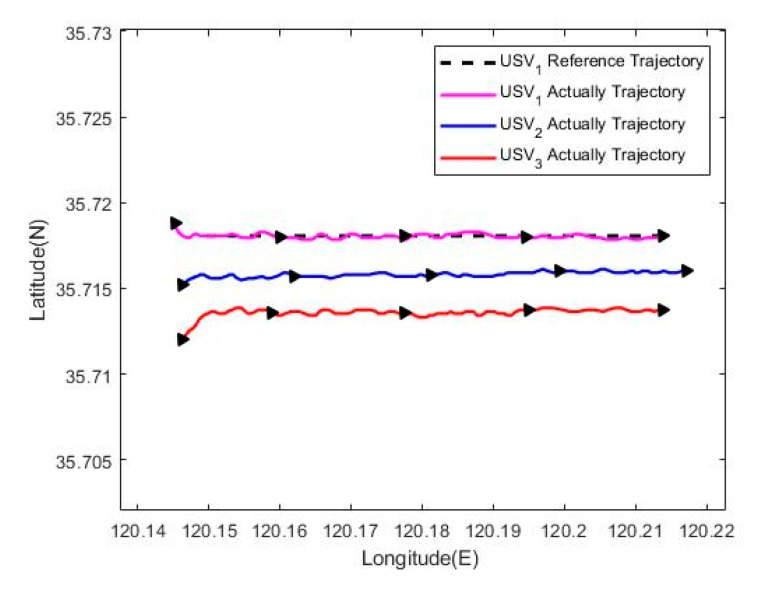
Tracking trajectory of the USV formation.

**Figure 33:**
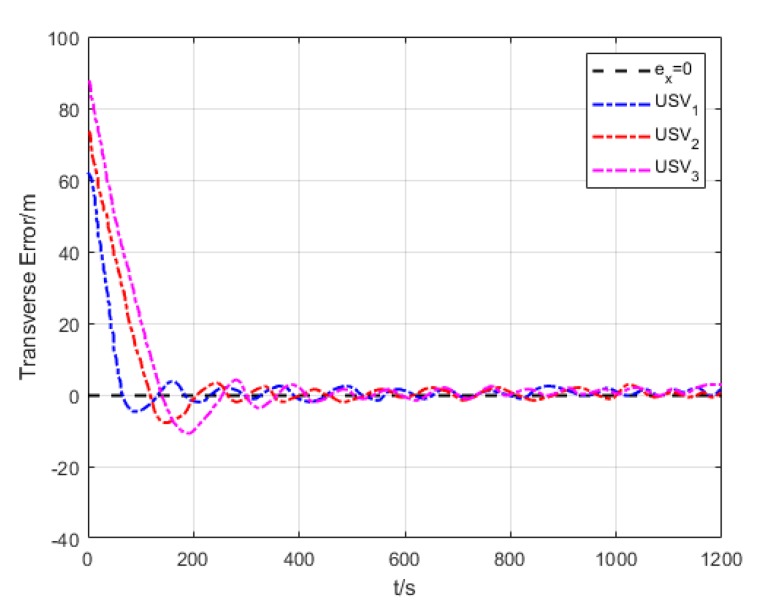
Variation of USV transverse error.

**Figure 34:**
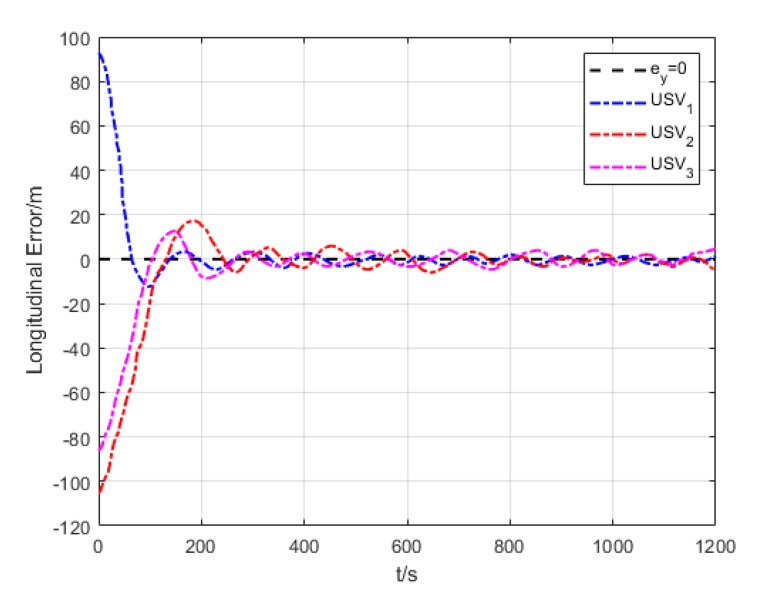
Variation of USV longitudinal error.

**Figure 35:**
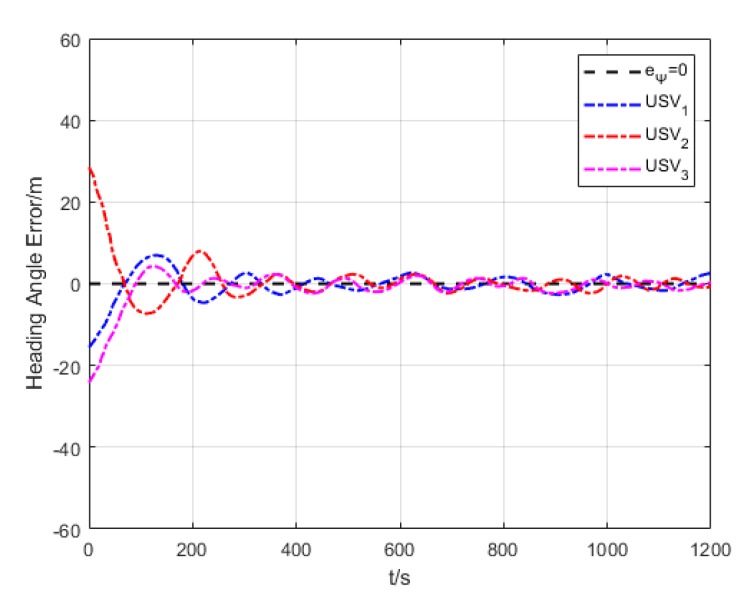
Variation of heading angle error.

**Figure 36:**
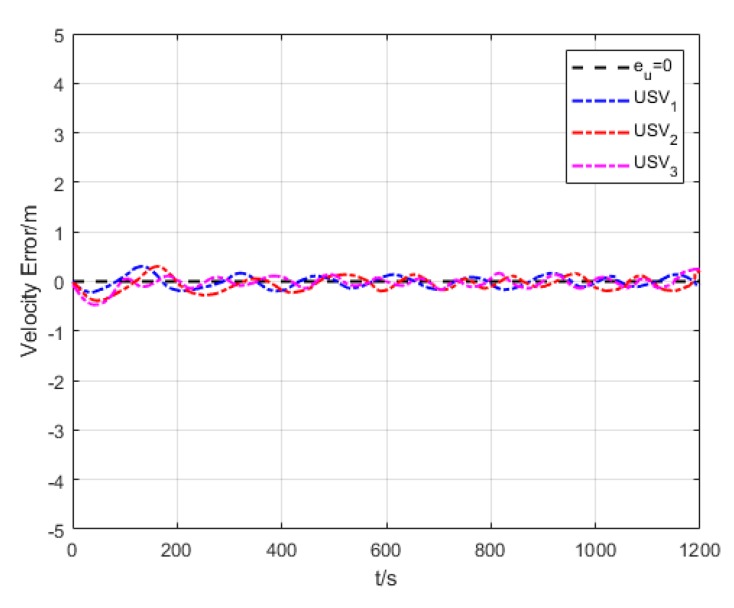
Variation of velocity error.

**Figure 37:**
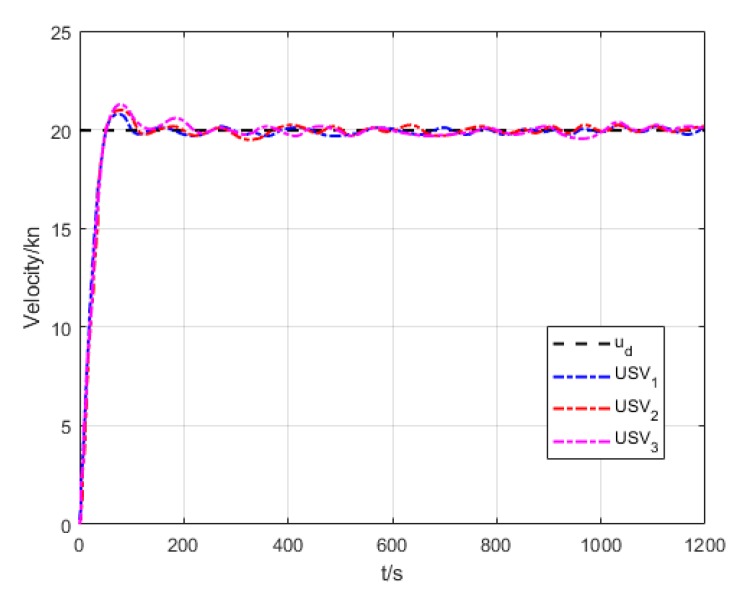
Variation of velocity.

The results of USV formation cruising control are shown in Figure 32, Figure 33, Figure 34, Figure 35, Figure 36 and Figure 37. Among them, Figure 32 presents the cruising track of USV formation, revealing that the proposed formation cruising control algorithm in this paper enables formation members gradually converge to the expected formation in the setting, and realizes the straight path tracking of the expected formation.

Figure 33 and Figure 34 present the variation curves of USV transverse and longitudinal position tracking errors in the process of formation cruising. As revealed, the formation control algorithm makes both the transverse and longitudinal errors of each USV converge fast to around 0 with lower oscillation. Meanwhile, each USV has the transverse error of around 3 m and the longitudinal error of around 4 m while maintaining the formation.

As shown in Figure 35, each USV has large oscillation amplitude of heading angle error while making the formation, and could converge fast to the expected heading angle, but smaller heading angle error while maintaining the formation (approximately 2°).

Figure 36 shows the velocity error curve of each USV in the process of formation cruising. It is revealed that each USV has large oscillation amplitude of velocity error while making the formation, and could also converge fast to around the expected velocity, but small velocity error while maintaining the formation (approximately 0.5 kn).

Figure 37 presents the velocity variation curve of each USV in the process of formation cruising. As we can see in the figure, velocity varies dramatically at the initial stage of making the formation, but each USV could stabilize the velocity around 15 kn to maintain the formation.

## 7. Conclusions

This paper proposes an improved ILOS guidance strategy while considering the influence of USV under-actuation, uncertainty, and external disturbance. A single USV path tracking controller is designed, while the idea of feedback control is followed to complete the stabilization of position error, heading angle, and expected velocity, so as to realize the single USV path tracking control. Based on the graph theory, a decentralized speed coordination controller is designed to realize USV formation tracking and maintain the formation. At last, cascade system theory and Lyapunov stability are utilized to respectively prove the uniform semi-global exponential stability of path tracking control system and the global asymptotic stability and uniform local exponential stability of coordinated formation system. Simulation and field experiment is conducted and analyzed to prove the advancement and effectiveness of path tracking algorithm and coordinated formation control algorithm based on the improved ILOS guidance strategy.

## Figures and Tables

**Table 1:** Model parameters of USV.

Model Parameter	Value	Model Parameter	Value
$m_{11}$	25.8	$b_{32}$	1
$m_{22}$	33.8	$d_{11}$	2.0
$m_{23}$	−11.748	$d_{22}$	7.0
$m_{32}$	−11.748	$d_{23}$	−2.5425
$m_{33}$	6.813	$d_{32}$	−2.5425
$b_{11}$	1	$d_{33}$	1.422
$b_{22}$	0		

**Table 2:** Initial parameters of USVs.

	x	y	ψ	u	v	r
${\mathrm{USV}}_{1}$	0	120	0°	0	0	0
${\mathrm{USV}}_{2}$	0	85	45°	0	0	0
${\mathrm{USV}}_{3}$	0	55	60°	1	0	0
${\mathrm{USV}}_{4}$	70	0	90°	2	1	0
${\mathrm{USV}}_{5}$	120	0	0°	2.5	1	0.5

**Table 3:** Initial parameters of USVs.

	Longitude	Latitude	ψ	u	v	r
${\mathrm{USV}}_{1}$	$120.144640^{\circ }E$	$35.7111377^{\circ }N$	137°	0	0	0
${\mathrm{USV}}_{2}$	$120.145960^{\circ }E$	$35.7051077^{\circ }N$	52°	0	0	0
${\mathrm{USV}}_{3}$	$120.145300^{\circ }E$	$35.7005077^{\circ }N$	43°	0	0	0
